# Core signalling motif displaying multistability through multi-state enzymes

**DOI:** 10.1098/rsif.2016.0524

**Published:** 2016-10

**Authors:** Song Feng, Meritxell Sáez, Carsten Wiuf, Elisenda Feliu, Orkun S. Soyer

**Affiliations:** 1 School of Life Sciences, University of Warwick, Coventry, UK; 2 Department of Mathematical Sciences, University of Copenhagen, Universitetsparken 5, 2100 Copenhagen, Denmark

**Keywords:** bistability, futile cycles, synthetic biology, signalling networks, competition

## Abstract

Bistability, and more generally multistability, is a key system dynamics feature enabling decision-making and memory in cells. Deciphering the molecular determinants of multistability is thus crucial for a better understanding of cellular pathways and their (re)engineering in synthetic biology. Here, we show that a key motif found predominantly in eukaryotic signalling systems, namely a futile signalling cycle, can display bistability when featuring a two-state kinase. We provide necessary and sufficient mathematical conditions on the kinetic parameters of this motif that guarantee the existence of multiple steady states. These conditions foster the intuition that bistability arises as a consequence of competition between the two states of the kinase. Extending from this result, we find that increasing the number of kinase states linearly translates into an increase in the number of steady states in the system. These findings reveal, to our knowledge, a new mechanism for the generation of bistability and multistability in cellular signalling systems. Further the futile cycle featuring a two-state kinase is among the smallest bistable signalling motifs. We show that multi-state kinases and the described competition-based motif are part of several natural signalling systems and thereby could enable them to implement complex information processing through multistability. These results indicate that multi-state kinases in signalling systems are readily exploited by natural evolution and could equally be used by synthetic approaches for the generation of multistable information processing systems at the cellular level.

## 1. Introduction

Cells sense environmental stimuli and use these to initiate appropriate physiological responses. Understanding such cellular information processing in healthy and diseased states [1–3], and engineering it through synthetic biology [4–7], requires better insights into the relationship between different interaction motifs found in signalling networks and their potential roles in the ensuing system dynamics [8]. To this end, a key interaction motif found predominantly in eukaryotic signalling systems is that of a futile signalling cycle, where a substrate protein is phosphorylated by a kinase and dephosphorylated by a phosphatase. When these enzymes are saturated by their substrate, this motif can display ultrasensitive response dynamics, enabling threshold responses to graded input signals [9]. It can also be shown theoretically, that the futile cycle motif in its simple form cannot enable bistability (see below). Experimental studies of cellular systems embedding the futile signalling cycle for several physiological responses, including cell fate determination and cell division [10–13], found ultrasensitive responses and in some cases bistability [14–21]. While the presence of bistability has been indicated to be functionally significant, for example, in the generation of phenotypic variability [22–25], its molecular implementations have not been fully elucidated.

To achieve bistability in a futile signalling cycle motif, the originally studied structure of this motif needs to be extended with additional features. Theoretical studies have shown that bistability can be achieved if there are feedback interactions between the substrate and its acting enyzmes (i.e. the kinase or phosphatase) [26–30], or if the substrate has multiple phosphorylation sites [31–34]. The latter proposition is particularly interesting as the presence of multiple phosphorylation sites on signalling proteins is a common phenomenon [35,36]. Kinases, phosphatases, as well as their substrates readily exhibit two or more conformational states that are associated with different levels of phosphorylation and that result in different catalytic activity levels [37–39]. In the signalling pathways regulating the cell cycle, for example, it has been hypothesized that signalling proteins with multiple phosphorylation sites act as multi-state enzymes that can embed complex signal processing [38–41]. It is also shown that the different activity levels of signalling proteins can be regulated through allosteric interactions with ligands or other proteins, such as the so-called scaffolding proteins [37,42–44]. Scaffolding proteins, which are ubiquitous in signalling systems [42,43], can also have multiple phosphorylation and binding sites themselves and, as such, are key regulators in signalling pathways [11,45–48]. Despite these experimental findings and observations on specific signalling proteins and pathways, it has been difficult to elucidate any particular features, or design principles, that can provide a clear understanding between the nature of signal processing that a system implements and the presence of multi-phosphorylation-site-featuring multi-state enzymes. This difficulty arises partially from the fact that modelling of signalling pathways with multi-state enzymes becomes increasingly complex, with a combinatorial explosion of possible interactions in the system.

Here, we perform a systematic, mathematical analysis of the effects of having multi-state kinases on the response dynamics and the number of steady states in a simple and core futile signalling cycle motif. When this motif is analysed with the assumption of single-state enzymes, the resulting system cannot display bistability for any positive kinetic parameter values. This situation changes and bistability becomes possible with the introduction of a two-state kinase, leading to one of the smallest signalling systems that is bistable and comparable in size to previously identified minimal systems [12,22,31–34,49–54]. Using this minimalist system as a tractable core motif, we are able to derive mathematical conditions on the kinetic parameters and/or the total concentrations of substrate and kinase that are necessary and sufficient for the existence of three steady states. This allows an intuitive insight that bistability in this minimalist system arises from the competition between the different states of the kinase for the substrate. Extending from this intuition, we show that increasing the number of kinase states in the system leads to a linear increase in the number of steady states. We show that both multi-state enzymes and the discussed core motif are prevalent in many signalling pathways and that the identified parameter ranges for bistability are biologically plausible. These results provide an intuitive view on multi-state enzymes leading to bistability and multistability through competition for their substrates. As such, the multi-state nature of enzymes can be exploited to better understand natural signalling pathways and to engineer novel ones.

## 2. Results

### 2.1. The futile signalling cycle with a two-state kinase is a bistable motif

**Figure 1.**
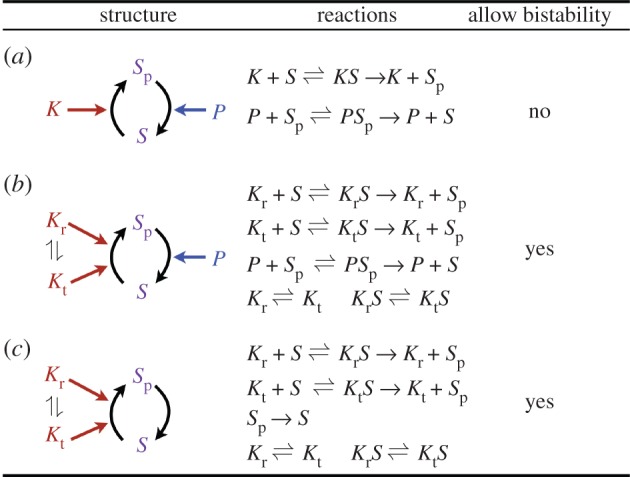
Cartoon of three signalling motifs. (*a*). A simple futile signalling cycle with a substrate that has a single phosphorylation site and is acted upon by a kinase and phosphatase. Both enzymes are assumed not to be allosteric. (*b*). The proposed bistable futile signalling cycle derived from (*a*). The substrate is phosphorylated by a two-state kinase and dephosphorylated by a single-state phosphatase. (*c*). The core signalling motif consisting of a futile cycle with a two-state kinase. The phosphorylated substrate undergoes auto-dephosphorylation at a constant rate.

A key interaction motif found in eukaryotic signalling networks is the so-called futile signalling cycle (figure 1*a*). When considered with a single phosphorylation site on the substrate and a simple, one-state kinase and phosphatase, this motif cannot display bistability for any choice of positive parameters (e.g. [34,55]). When we extended this system with a two-state kinase, this key result changed and bistability was possible. We introduced the two-state kinase such that each state can bind the substrate and catalyse its phosphorylation, and where transitions between the two states are possible irrespective of substrate binding (figure 1*b*). The two-state kinase in this simple model switches between two conformational states with a constant rate. The two states show differential catalytic activity towards the substrate (figure 1*b*, Material and methods). While this is the simplest model to introduce the idea of multi-state enzymes into the core futile cycle motif, it is readily possible to assume more complex models. In particular, the conformational change between kinase states can be modelled as an allosteric regulation [56–60], whereby it is linked to binding of the kinases by a ligand or other proteins, or as arising from covalent phosphorylation events as commonly observed in signalling proteins [26,36,61]. We consider such complex models below, but note that adding this complexity does not alter the key conclusions of this study on bistability and multistability.

We find that the core motif with two-state kinases can be further simplified without compromising bistability by removing the phosphatase and letting the dephosphorylation of the substrate happen through auto-hydrolysis at a constant rate (figure 1*c*, Material and methods). In this way, we obtain a minimal core signalling system driven by a two-state kinase, which displays bistability. The system contains only six species, making it one of the smallest bistable signalling motifs.

### 2.2. Conditions for bistability in the core motif are satisfied in a biologically plausible range

The simplicity of this core motif allowed us to analytically study the solutions to the steady-state equations (see the electronic supplementary material). In particular, we were able to derive a set of inequalities in the kinetic parameters and total concentrations of the substrate and kinase that provide a set of necessary and sufficient conditions for the existence of three steady states in the system (equation (4.5), see also the electronic supplementary material for the derivation of this equation). While equation (4.5) provides a necessary and sufficient condition on parameters for the presence of multistationarity, we also derive a simpler equation representing the necessary condition for bistability:
2.1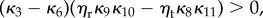;where the indexing of the rate constants is as given in equation (4.1) in Material and methods, and the composite parameters 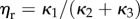 and 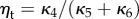 are the inverses of the Michaelis–Menten constants of *K*_r_ (the kinase at the relaxed state) and *K*_t_ (the kinase at the tense state), respectively. Analysis of this equation reveals some of the key structural features of the system that are necessary for bistability, in particular: (i) the conversion between the two free forms of the kinase and (ii) the conversion between the two substrate-bound forms of the kinase. That is, both *κ*_8_ and *κ*_9_ cannot be zero, and both *κ*_10_ and *κ*_11_ cannot be zero. Thus, the structure of the reaction system comprised of a futile signalling cycle driven by a two-state kinase is crucial for enabling bistability.

**Figure 2.**
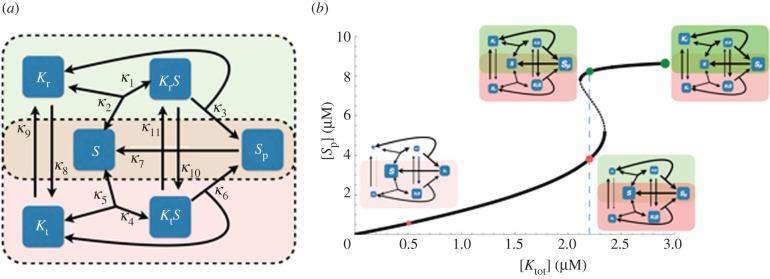
Schematic of the core signalling motif displaying bistability. (*a*) Cartoon representation of the two interconnected reaction cycles constituting the core bistable system. The arrows represent reactions in the system and are labelled with the kinetic parameters from equation (4.2). Two rectangles (dashed line) with different background colour show the two futile cycles with *K*_r_ (green) and *K*_t_ (red) competing for the substrate (in the intersected region of the two rectangles). (*b*) Bifurcation plot of core bistable signalling motif. The solid line corresponds to the stable steady-state levels of [*S*_p_] with increasing signal given by the total concentration of kinase [*K*_tot_]. The dashed line corresponds to the unstable steady states. The parameter values used to generate the bifurcation plot are listed in the electronic supplementary material, table S3. The four little cartoons, drawn as inset, are showing the allocation of all species' concentrations and corresponding reaction fluxes at the different levels of [*K*_tot_], as indicated by the coloured dots. For each cartoon, the size of the species' boxes and reaction arrows are calculated from the actual species' concentrations and the levels of the reaction fluxes, namely *κ*_1_[*K*_r_*S*], *κ*_2_[*K*_r_*S*], *κ*_3_[*K*_r_*S*], *κ*_4_[*K*_t_*S*], *κ*_5_[*K*_t_*S*], *κ*_6_[*K*_t_*S*], *κ*_7_[*S*_p_], *κ*_8_[*K*_r_], *κ*_9_[*K*_t_], *κ*_10_[*K*_r_*S*] and *κ*_11_[*K*_t_*S*]. Specifically, the species' concentrations and reaction flux values are mapped on the cartoon by log-transforming the actual value and then mapping these onto a specific interval; the intervals used for arrow thickness and box size were [0.5,13] and [20,100], respectively. The transparency index for the two overlay boxes indicating two futile cycles mediated by *K*_r_ (green) and *K*_t_ (red) are calculated by log-transforming the total concentrations of *K*_r_ and *K*_t_ containing species and mapping these onto an interval [0,100].

Furthermore, equation (2.1) provides two key dynamical features for bistability. Firstly, the two interconnected futile cycles between *S* and *S*_p_, defined by the two kinase states, need to operate at different catalytic rates (i.e. *κ*_3_ ≠ *κ*_6_). Secondly, the switching between these cycles through the four forms of the kinase (i.e. *K*_r_, *K*_t_, *K*_r_*S*, *K*_t_*S*) needs to occur at different rates, and in a way opposing the difference in the catalytic rates. Specifically, if the futile cycle for the relaxed state of the kinase (i.e. *K*_r_ and *K*_r_*S*) has the highest catalytic activity (i.e. *κ*_3_ > *κ*_6_), then *η*_r_*κ*_9_*κ*_10_ needs to be larger than *η*_t_*κ*_8_*κ*_11_. As a consequence, the clockwise interchanging cycle, 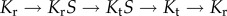, corresponding to the product of the rate constants 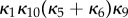, needs to dominate over the anti-clockwise cycle, 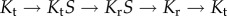, corresponding to the product 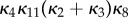. Symmetrically, if *K*_t_ has higher catalytic activity than *K*_r_ (i.e. *κ*_3_ < *κ*_6_), then the anti-clockwise cycle needs to dominate (see also figure 2 and discussion below).

A further constraint on the rates governing the transitions among the four forms of the kinase might arise from thermodynamics. Particularly, these transitions form a local reaction cycle, which must follow the principle of detailed balance if we assume no additional energy input into the system [62–65]. This results in a thermodynamic constraint on the reaction kinetics such that the product of the rate constants in the clockwise direction must equal the product of the reverse rate constants (i.e. *κ*_1_*κ*_5_*κ*_9_*κ*_10_ = *κ*_2_*κ*_4_*κ*_8_*κ*_11_). It must also be noted, however, that this constraint would be relaxed if the conformational switching between the enzyme states were directed by energy input (e.g. phosphorylation–dephosphorylation reactions; electronic supplementary material, figure S1*a*) or steric effects with enzyme binding with other proteins or enzymes (electronic supplementary material, figure S1*b*).

To determine whether these conditions on kinetic rates can be simultaneously satisfied in cellular signalling networks, we tabulated kinetic parameters from the literature (see the electronic supplementary material, table S1 and references therein). We then sampled 10^5^ parameter sets around these known kinetic parameters and checked whether the necessary and sufficient conditions for bistability were satisfied (see Material and methods). This analysis showed that the futile signalling cycle displays bistability in a biologically plausible parameter regime, even when thermodynamic constraints are taken into account (electronic supplementary material, figure S2 and table S2).

### 2.3. Bistability can be seen as arising from competition between the kinase states for the substrate and resulting positive feedback loops

It is interesting to note that the mathematical conditions derived in equation (2.1) impose a specific structure onto the core motif. As discussed above, this comprises the conversion between the four, free and substrate-bound forms of the kinase. The interactions of these different kinase states with the substrate can be seen as two connected reaction cycles involving competition for the same substrate (figure 2*a*). Equation (2.1) shows that the flows of these two competing reaction cycles need to have a specific relationship for bistability to emerge. To better understand these ensuing reaction fluxes, we have analysed the steady states of the system for increasing total kinase concentration, as a proxy for an increasing signal (figure 2*b*, see also the electronic supplementary material, sections 1.8 and 1.9). For a fixed set of parameters in the bistable regime such that *κ*_3_ > *κ*_6_, *κ*_9_ > *κ*_8_, *κ*_10_ > *κ*_11_ and *η*_r_*κ*_9_*κ*_10_ > *η*_t_*κ*_8_*κ*_11_ (see the electronic supplementary material, table S3), we find that in the low signal regime, where the total level of kinase is low, there is a large flux from *K*_r_*S* into *K*_t_*S*, resulting in the accumulation of *K*_t_*S*. Thus in this low signal regime, the slow futile cycle driven by *K*_t_ (which has the lowest catalytic activity) dominates (i.e. [*K*_r_] + [*K*_r_*S*] < [*K*_t_] + [*K*_t_*S*]) and the system is at ‘low’ state (i.e. small [*S*_p_]) (figure 2*b*, red dots). In the high signal regime, the fast futile cycle driven by *K*_r_ dominates (i.e. [*K*_r_] + [*K*_r_*S*] > [*K*_t_] + [*K*_t_*S*]) and the system is at the ‘high’ state (i.e. large [*S*_p_]). The substrate is largely converted to the phosphorylated form, which results in the accumulation of *K*_r_ (figure 2*b*, green dots). Whether the *K*_r_-mediated or *K*_t_-mediated cycle dominates is determined by the interplay between the catalytic constants, the Michaelis–Menten constants associated with each kinase form, and the transition rate constants between these forms in a free and substrate-bound state.

This analysis derived from the necessary parameter condition (equation (2.1)) leads to an intuitive view, in which the bistability in the system is understood as a result of the two futile cycles driven by the two forms of the kinase competing for the substrate. Furthermore, the competing kinase forms need to have opposite dominance in terms of being able to bind the substrate and their catalytic activity, such that the form dominating catalytically needs to be weaker in terms of substrate binding kinetics (after correction by the transition rate constants). For example, if *κ*_3_ > *κ*_6_, then we need to have *η*_r_*κ*_9_*κ*_10_ > *η*_t_*κ*_8_*κ*_11_. Interestingly, this motif structure embedding competition between the kinase forms for the substrate also gives rise to positive feedback loops [66] that are not readily seen from the reaction cartoon, but can be found within the bipartite graph that encodes how the species and reactions of the network influence each other (see the electronic supplementary material, SI 1.10).

### 2.4. Increasing the number of kinase states in the futile cycle increases the number of steady states (theoretically unbounded multistationarity)

Recognizing that bistability in the core motif is linked to the competition between the two futile cycles, it is intriguing to consider whether adding more competing cycles increases the number of steady states. To expand from the simplest motif towards more complicated systems, one way of increasing competing cycles is to increase the number of two-state kinases, while the other is to increase the number of states of a single kinase. We find that both expansions of the minimal system result in an increase of the number of steady states.

**Figure 3.**
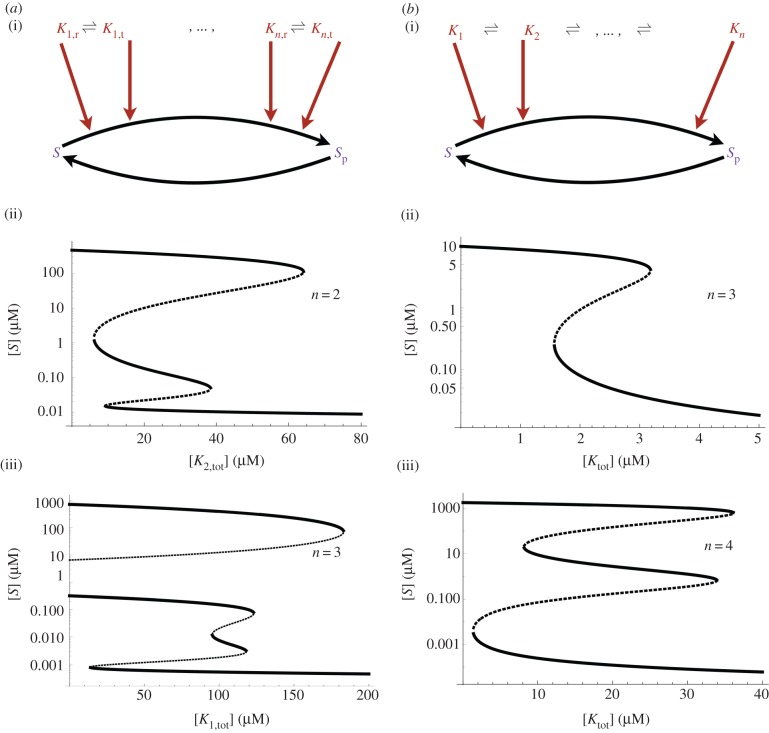
Implementation of multistability by expanding the core bistable motif. (*a*) Multistability generated by a signalling cycle with multiple two-state kinases. (i) A schematic of the core bistable motif extended with multiple allosteric kinases. (ii) Bifurcation plot for a system with two allosteric kinases. The *x*-axis shows the signal level [*K*_2,tot_] (total concentration of the second kinase *K*_2_), while the *y*-axis shows the steady-state level of [*S*] (unphosphorylated substrate). (iii) Bifurcation plot of a system with three allosteric kinases. The *x*- and *y*-axes are as above. Parameter values used for the bifurcation plots are listed in the electronic supplementary material, table S4. (*b*) Multistability generated by a signalling cycle with a multi-state kinase. (i) Schematic of a multi-state kinase catalysing a futile signalling cycle. (ii) Bifurcation plot of a system with a three-state kinase. (iii) Bifurcation plot of a system with a four-state kinase. The *x*- and *y*-axes are as above. In all bifurcation plots, solid lines correspond to stable steady states, while dashed lines correspond to unstable steady states. All axes use the unit of concentration micromolar. Parameter values used for the bifurcation plots are listed in the electronic supplementary material, table S5.

Firstly, we considered the case of multiple kinases with two states (figure 3*a*). In this case, multiple two-state kinases in a futile cycle lead to multistationarity (figure 3*a*). With the number of kinases *n* increasing, the number of steady states linearly scales with *n*. We prove that the system can admit at most 2*n* + 1 steady states and further that *n* of them are unstable (see the electronic supplementary material). The other *n* + 1 steady states are presumably stable. Secondly, multistability can be achieved by one kinase with multiple states (figure 3*b*). When the kinase has three distinct states, the system can have three steady states at most, but a four-state kinase results in the possibility of five steady states at most (figure 3*b*; see the electronic supplementary material). The general scenario with an *n*-state kinase is too complex mathematically and does not admit the approach used to analyse systems with multiple two-state kinases. However, we make the conjecture that the number of positive steady states grows linearly with *n* as well, such that the system admits at most *n* + 1 positive steady states if *n* is even and *n* positive steady states if *n* is odd.

### 2.5. Multistability enables complex state transitions

The above results confirm that a single futile signalling cycle with a two-state kinase can generate bistable dynamics and that such a system can be expanded by increasing the number of kinase states to achieve unlimited multistationarity. In this scenario, each additional kinase state potentially drives the generation of a pair of steady states, one stable and one unstable, due to the competition for the substrate. Thus, it should be possible to use the total concentrations (or kinetic parameters) of the different kinases to change the signal thresholds to switch between steady states and implement logic gates in this way. More specifically, in the system with multiple two-state kinases, varying the total concentration of a kinase can dictate the system transitions among the different steady states resulting from multistability.

**Figure 4.**
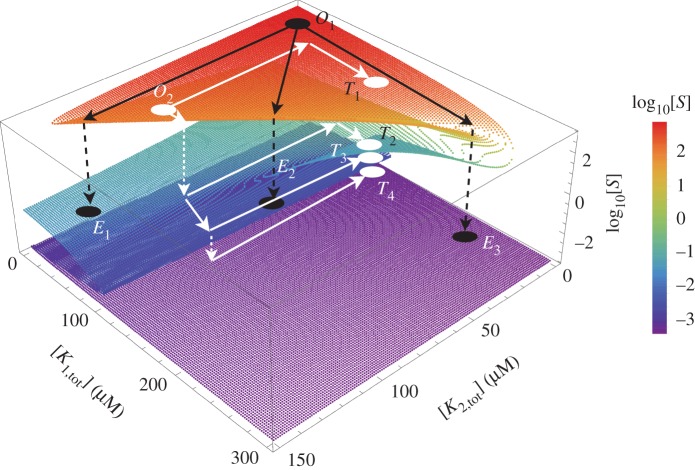
Multistability installs complex state transitions. The steady-state level of the unphosphorylated substrate, [*S*], for different levels of the two kinases, [*K*_1,tot_] and [*K*_2,tot_]. The colour-coding shows the level of unphosphorylated substrate, [*S*], for each amount of kinase. The black and white dots represent specific states of the system. The black and white arrows show the hypothetical trajectories described when the kinase levels are perturbed in various combinatorial ways, as discussed in the main text.

Here, we show that by combinatorial perturbations of different kinases, a system with three two-state kinases can perform complex state transitions (figure 4). The varying parameters are the total concentrations of the first two kinases, namely [*K*_1,tot_] and [*K*_2,tot_]. We assume that the system starts off at a given state (*O*_1_ in figure 4) with low total concentration of both kinases. By increasing the total concentration of either kinase (*K*_1_, *K*_2_) or both, the system can be made to switch to three different end-states of [*S*] (figure 4, points *E*_3_, *E*_1_ or *E*_2_). It is also possible to bring the system into different states by perturbing the total concentrations of both kinases by a fixed amount each, but following different sequential moves (figure 4, from *O*_2_ to *T*_1_, *T*_2_, *T*_3_ and *T*_4_). In these examples, the final system output is a function of the combinatorial activity patterns of both kinases. By contrast, different perturbations would result in the same output state in a monostable system. Therefore, multistability can encode the specific order of changes in the environmental signals (i.e. different kinase activities) into different system outputs at steady state. The result is a potential increase in the system's capacity to store information, e.g. relating to fluctuating or complex environments.

### 2.6. Natural signalling pathways display complex interactions leading to multi-state enzymes and potential for multistability

**Figure 5.**
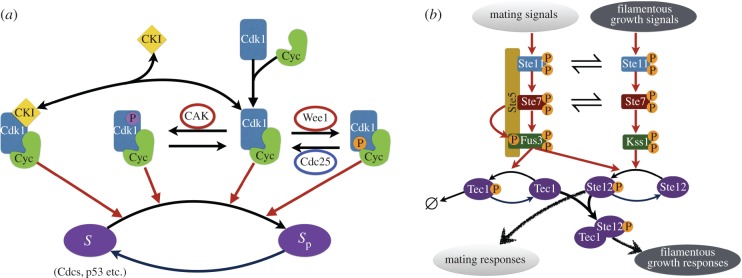
The bistable signalling motif in biological systems. (*a*) Different forms of regulation of Cdk1's catalytic activity give rise to different states of Cdk1. The multiple states of Cdk1 are involved in catalysing many downstream substrates, including Cdc and p53. Such catalytic reactions show precisely the structural pattern in figure 3*b*. (*b*) The two MAPK cascades in yeast mating response and filamentous growth response. The two cascades share Ste11 and Ste7. Ste11, Ste7 and Kss1 have two phosphorylation sites while Fus3 has three phosphorylation sites, one of which is phosphorylated by Ste5. This schematic shows that in both cascades all three layers of signalling enzymes, MAP3 K (i.e. Ste11), MAP2 K (i.e. Ste7) and MAPKs (i.e. Fus3 and Kss1) exhibit different states that compete for their substrates. Thus, the cross-talk between the two cascades and the presence of the scaffold protein increases the number of states of the enzymes, resulting in a system similar to that considered in figure 3*a*.

As discussed in the Introduction, futile signalling cycles are ubiquitous motifs in natural signalling networks, where they feature multi-state enzymes. To demonstrate this point, we explore two example cases of natural signalling cycles. The first example comprises the signalling networks controlling the cell cycle, in particular networks involving cyclin-dependent kinases (Cdks). It is argued that the activity of Cdks is a key mechanism for ensuring appropriate switching dynamics for the cell cycle [38–40]. The activity level of Cdk1 is regulated by four different mechanisms: (i) activating phosphorylation by Cdk-activating kinases (CAKs), where phosphorylation by a CAK of a threonine residue increases the kinase activity of Cdk1 [67]; (ii) inhibitory phosphorylation by Wee1, where phosphorylation of a tyrosine residue by Wee1 reduces kinase activity of Cdk1 [68]; (iii) cyclin binding, where cyclins binding cooperatively to Cdk1 and their substrates promote Cdk1 kinase activity [69]; and (iv) Cdk-inhibitor (CKI) binding, where CKIs bind to Cdk1 and block their active sites [68] (figure 5*a*). Such combinatorial interactions (i.e. regulations) where different Cdk1 ‘states’ (i.e. phosphorylated at different positions, bound/unbound, etc.) compete for the same downstream substrates with potentially different activity levels, can thus create a situation similar to that shown in figure 3*b*. It must also be noted that there are multiple homologous Cdks in the cell [41,68], which would replicate this situation of multi-state enzymes competing for the same substrates to result in a picture as shown in figure 3*a*. The system is further complicated with the multi-state nature of the enzymes upstream of Cdk's. For example, Wee1 has differentially phosphorylated forms that can have different activity towards Cdk1 [70,71]. The second example for multi-state enzymes comes from the MAPK signalling cascades [32]. For instance, the MAPK signalling networks controlling yeast mating response and filamentous growth response share the signalling proteins Ste11 and Ste7, both of which have two phosphorylation sites and can bind to a scaffolding protein Ste5 (figure 5*b*) [44]. The possible combinatorial interactions and the different phospho-states of these proteins, as well as their downstream interaction partners such as Fus3 and Kss1, provide a system with multiple kinase states.

The picture emerging from the Cdk-based cell cycle as well as the MAPK pathways is one with several multi-state enzymes in competition for the same substrates. This picture fits in the simplified motifs as analysed above, making it theoretically possible for these pathways to display bistability and multistability. For this possibility to be realized, the different states of the key signalling enzymes in the natural pathways must show different binding and catalytic activity levels towards their substrates as mathematically analysed above. The regulation of transitions among such states would then be expected to form an important aspect of cellular dynamics and information processing. While this viewpoint fits with some of the current knowledge, for example, different Wee1 forms having different activity levels, with some being indicated as ‘inactive’ [70,71], and ubiquitination-based control of Wee1 being critical in cell cycle progression [72], full experimental verification of the relationship between cellular information processing and the presented theory of multistability requires further studies. A critical starting point would be measuring *in vitro* the catalytic and binding rates of different enzyme forms found in these systems to see if they fit with the mathematical conditions for multistability presented here.

## 3. Discussion

The key finding of this study is that the presence of a multi-state kinase in the common futile signalling cycle motif allows this functional interaction system to display bistability. Thus, a phosphorylable substrate with a two-state kinase forms one of the smallest bistable signalling motifs. The emergence of bistability in this simple system relates closely to the two states of the kinase forming two futile cycles that are competing for the substrate. We define conditions on the kinetic parameters of these two competing cycles that are necessary and sufficient for three steady states. We show that these conditions are met under biologically feasible parameter regimes. Finally, we find that increasing either the number of two-state kinases acting on the same substrate or the number of distinct states that a single kinase can exhibit increases the number of steady states in an unbounded manner.

The core bistable signalling motif featuring multi-state enzymes is prevalent in biological systems. The presence of multiple conformational states with differential activity is a common feature of many enzymes [57], and particularly in signalling networks, where many kinases and phosphatases admit multiple states that display different levels of activity and that are regulated through covalent modification or interaction with scaffold proteins [42,73]. As we have shown above, using Cdks and MAPK pathways as examples, there are several natural cases where such interactions create or embed the described core bistable motifs or extensions of it. Our findings thus provide mathematical proof that these natural systems can theoretically allow bistability and potentially unbounded multistability. Transitions between the steady states can underpin the capacity of cells to map environmental states to internal gene expression and physiology, increasing their ability to adapt to different or fluctuating environments. The validation and further interrogation of these possibilities must come from experimental studies. In particular, synthetic biology approaches can be used to implement the core bistable motif described here using existing multi-state proteins and kinases from nature and analysing their dynamics in a controlled manner. These approaches are already being employed to study MAPK and two-component signalling systems [73–76], and can be further extended using the presented results as guiding principles for experiments.

An intuitive interpretation of our results is that competition of different futile cycles for the same substrate is a key prerequisite for bistability. This intuitive view can also be applied to understand previously described bistable and multistable signalling motifs. For instance, a substrate with multiple phosphorylation sites that are acted upon by the same kinase is shown to implement bistability and multistability [31–34,77]. This system is almost a symmetric version of the system we consider here, as it features futile cycles involving differently phosphorylated substrates competing for the same enzyme. Another example of a bistable system is where a futile cycle can take place in two different compartments, with both substrates and enzymes shuttling between the two compartments. This again fits our intuitive view, where the separation of enzymes and substrates in different compartments creates a set of futile cycles that are competing for both substrates and enzymes [54].

These examples indicate that competing futile cycles could provide a general condition for determining bistability. In order to validate this idea, further exploration of different motifs and the structural conditions on multistationarity is required. One possible approach would be to enumerate a large set of small signalling networks and compare structural differences between monostationary and multistationary networks. Specific structural patterns might emerge, which can be validated by further mathematical analyses. These mathematically derived conditions can then be used to better understand natural signalling systems and design bistable signalling networks and biochemical memory through synthetic biology.

## 4. Material and methods

### 4.1. Core model for a futile signalling cycle with a two-state kinase

The core futile signalling cycle we consider here consists of a covalent modification, i.e. de/phosphorylation, of a substrate by a kinase and a phosphatase [9,78]. We extend this motif by considering multiple possible states of the kinase that could potentially have differing activity levels. For the case of the two-state kinase, we consider two distinct states (relaxed, *K*_r_, and tense, *K*_t_) catalysing a substrate (*S*) into a phosphorylated product (*S*_p_). To simplify the system, we do not model the phosphatase directly, but rather consider an auto-dephosphorylation reaction. The set of reactions we consider include the transformations between the different kinase states and Michaelis–Menten mechanisms for the phosphorylation of the substrate:
4.1
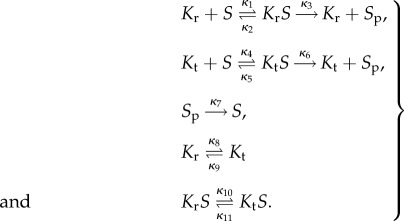
;The parameters *κ*_1_ to *κ*_11_ represent the kinetic parameters, also called rate constants. The system consists of six species, two of which are substrate–enzyme complexes. Assuming the law of mass action, we model the rate of change of the concentrations of each of the species with the following system of ordinary differential equations:
4.2
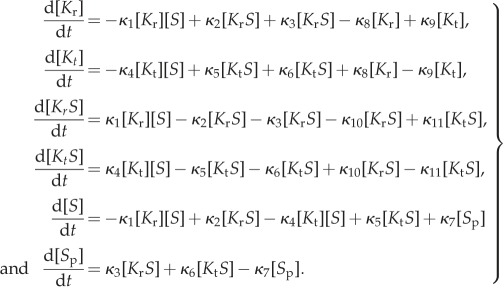
;

The system satisfies the following two conservation laws:
4.3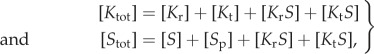;where we introduce two concentration invariants, namely [*K*_tot_] and [*S*_tot_], representing the total concentration of the kinase and the substrate, respectively. These equations arise from the biological assumption, implicit in the choice of reactions, that the total concentrations of the signalling proteins are constant over the relevant timescales of signalling (i.e. the model does not consider dynamics arising from gene regulation and expression).

### 4.2. Mathematical analysis of the steady states of the core motif

We consider the steady-state equations (obtained by setting the differential equations in equation (4.2) to zero) and the conservation law for [*K*_tot_] to express the steady-state concentrations of all variables in terms of the concentration of the unphosphorylated substrate (see the electronic supplementary material). Using the conservation law for [*S*_tot_], we obtain then a polynomial, whose positive solutions are the steady-state levels of [*S*] (see the electronic supplementary material, equation (9)). The coefficients of the polynomial depend on the rate constants and the total concentrations. We show that the polynomial has one, two or three positive roots (depending on the parameters), which proves that the core motif admits three steady states for a proper choice of rate constants and total concentrations of *S* and *K* ([*S*_tot_] and [*K*_tot_], respectively). We further show that one of the three steady states is unstable. Simulations show that the other two steady states are always stable.

We can further derive analytical conditions for the polynomial to have three positive roots, resulting in necessary (but not sufficient) conditions for bistability that depend on both the total concentrations and the rate constants:
4.4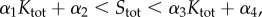;where *α*_1_, *α*_2_, *α*_3_ and *α*_4_ are positive expressions in the rate constants (see full expression in the electronic supplementary material, equation(10)).

Following an alternative approach, based on Brouwer Degree Theory (see [79,80], where the strategy was applied to study a multi-site phosphorylation system), we derive sufficient and necessary conditions on the 11 rate constants alone, as discussed in the Results, under which the system has three steady states (for the complete proof, see the electronic supplementary material, proposition 4). Namely:
4.5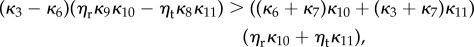;where 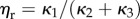 and 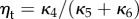 are the inverse of the Michaelis–Menten constants of the two conformational states of the kinase. While equation (4.5) presents the sufficient and necessary conditions on the 11 rate constants alone, we show in the electronic supplementary material, that if the parameters satisfy this equation, then there exists total concentrations [*S*_tot_] and [*K*_tot_] that result in three steady states in the system (see the electronic supplementary material, proposition 6).

Necessary and sufficient conditions for three steady states involving both the rate constants and the total concentrations can also be found for this system (see the electronic supplementary material, proposition 6). The expressions we obtain are complex and do not allow for easy biological interpretation. A guide on how to generate parameters that yield to three steady states is given in the electronic supplementary material (section 1.7).

### 4.3. Proof of unbounded multistability

The results for the extended models with *n* two-state kinases competing for the same substrate, or a single kinase with multiple states are detailed in the electronic supplementary material, sections 2 and 3. There, we show that there are rate constants for which the system with *n* allosteric kinases has exactly 2*m* + 1 (where *m* = 0, … ,*n*) positive steady states. This holds also for *m* = *n,* in which case there are 2*n* + 1 positive steady states. In fact, we show that 2*n* + 1 is the maximal possible number of steady states, positive as well as steady states for which at least one concentration is zero. To show these results, we follow the model reduction techniques from [81,82]. These techniques enable us to conclude the existence of multiple steady states for the networks of interest, from their existence for simpler networks that allow for a detailed mathematical analysis.

### 4.4. Checking bistability

We used the Chemical Reaction Network Theory Toolbox (CRNT Toolbox 2.3, https://crnt.osu.edu/CRNTWin) to analyse the networks shown in figure 1 and the electronic supplementary material, figure S1. The toolbox enables us to determine whether a (bio)chemical reaction network can have at least two steady states (for some choice of parameters) solely based on the structure of the network, assuming all reactions follow mass-action law. For each of the networks in figure 1 and electronic supplementary material, figure S1, we obtained a set of parameters for which the network admits at least two steady states. We then checked that, in each case, there were actually three steady states, two of which were stable and one unstable. For an introduction to the toolbox, we refer the reader to the manual that is downloaded with the software.

### 4.5. Parameter sampling

To sample from the parameter space of the rate constants, we assume ln *κ_i_* follows a uniform distribution, ln κ_*i*_ ∼ *U*(*a*, *b*), (*i* = 1, … ,11). We use *a* = ln 10^−3^ and *b* = ln 10^3^, based on biologically feasible parameters validated in experiments [83] (electronic supplementary material, table S1).

To sample a parameter set without the thermodynamic constraint, we simulate 11 uniform variables *u_i_*, *i* = 1, … ,11 with common distribution *U*(0, 1) and define *κ*_i_ = *e*^(*b* − *a*)*u*_*i*_ + *a*^. To sample rate constants with the thermodynamic constraint, we use a different approach. Under the constraint, two products of four kinetic rates are identical *κ*_1_*κ*_5_*κ*_9_*κ*_10_ = *κ*_2_*κ*_4_*κ*_8_*κ*_11_, which translates into an equality of two sums of logarithmic rates. We first assume the logarithmic rates lie between 0 and 1, and then afterwards transform them such that they lie between *a* and *b*. We follow three steps.

*Step 1*. In this step, we simulate the sum of the logarithmic rates using rejection sampling. To simulate the sum, we simulate four uniform variables *u*_1_, *u*_2_, *u*_3_, *u*_4_, with distribution *U*(0,1) and define *s* = *u*_1_ + *u*_2_ + *u*_3_ + *u*_4_. To take into account the constraint, the value *s* is accepted with the probability *p*(*s*) that the sum of the other four logarithmic rates takes the same value. This probability is derived from the Irwin–Hall distribution [84,85], and takes the form:
4.6
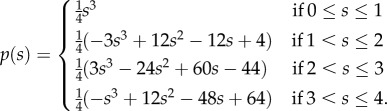
;

The probability might attain any value between 0 and 1. A random number *z* is then drawn from a uniform variable *Z* ∼ *U*(0,1). If *z* < *p*(*s*), we accept *s*. If not, we discard *s,* redraw *u_i_*, *z* and recalculate *p*(*s*) until *s* is accepted. We do this as many times as we want parameter sets.

*Step 2*. In this step, we simulate the individual logarithmic rates that sum up to the accepted values from the first step. For each accepted *s*, we simulate four uniform variables *U*(0, 1). Denote the variables by *p_i_*, *i* = 1, … ,4, and let their sum be *P.* Define 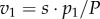, 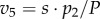, 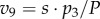, 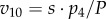, such that *v*_1_, *v*_5_, *v*_9_ and *v*_10_ add up to *s*. If *v*_1_, *v*_5_, *v*_9_, *v*_10_ are all less than one, accept them. Otherwise repeat the procedure by redrawing four new uniform variables until the condition is fulfilled. Next we do the same to obtain 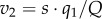, 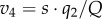, 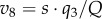, 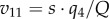, where *q_i_*, *i* = 1, … ,4, are uniform *U*(0,1) random variables with sum *Q*.

*Step 3*. We generate three random numbers *v*_3_, *v*_6_ and *v*_7_ from a uniform distribution *U*(0, 1). Finally, we compute the rate constants as 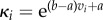, *i* = 1, … ,11.

Note that without the thermodynamic constraint, all rate constants are generated from the same distribution. With the constraint, *κ*_3_, *κ*_6_ and *κ*_7_ follow the same distribution as without the constraint. The remaining eight parameters follow a different distribution, as they are constrained. This distribution is common to all eight parameters as the equation for the constraint is symmetric in these parameters.

## Supplementary Material

Supplementary Information

## Supplementary Material

Supplementary Figure

## Supplementary Material

Supplementary Figure
