# Supplementary material for: Core signalling motif displaying multistability through multi-state enzymes
Source: J R Soc Interface. 2016 Oct;13(123):20160524. doi: 10.1098/rsif.2016.0524 (PMC5095215; doi:10.1098/rsif.2016.0524)
Supplement: Supplementary Information [file rsif20160524supp1.pdf]

Core signalling motif displaying multistability through allosteric enzymes

Song Feng, Meritxell Sáez, Carsten Wiuf, Elisenda Feliu and Orkun S Soyer

Supplementary Information

Mathematical explanations and proofs of the results in the main text

August 22, 2016

Contents

1 A model for an allosteric kinase 2
1.1 Model description . . . 2
1.2 Summary of results . . . 3
1.3 Model reduction and multistationarity . . . 4
1.4 Positive steady states . . . 5
1.4.1 The steady states as functions of [S] . . . 5
1.4.2 The steady state polynomial . . . 6
1.5 Necessary conditions for multistationarity . . . 7
1.6 Necessary and sufficient conditions for multistationarity . . . 8
1.6.1 Conditions involving only reaction rate constants . . . 8
1.6.2 Conditions involving reaction rate constants and total amounts . . . 10
1.7 Necessary and sufficient conditions in practice . . . 12
1.8 Describing the steady states . . . 14
1.8.1 The concentration $[S_p]$ as a function of $[S]$ . . . 14
1.8.2 The concentrations $[K_r]$ and $[K_t]$ as functions of $[S]$ . . . 16
1.8.3 The concentrations $[K_rS]$ and $[K_tS]$ as functions of $[S]$ . . . 17
1.9 Bifurcation plots with K_{tot} and S_{tot} . . . 18
1.9.1 Changing S_{tot} . . . 19
1.9.2 Changing K_{tot} . . . 21
1.10 Positive feedback loops . . . 24

2 The core model for n allosteric kinase competing for the same substrate 25
2.1 Model description . . . 25
2.2 Summary of results . . . 26
2.3 Positive steady states . . . 26
2.4 Existence of $2n+1$ positive steady states. . . . 27
2.5 n unstable steady states . . . 30

3 Allosteric kinases with several states 31

1 A model for an allosteric kinase

1.1 Model description

We consider a reaction network consisting of an allosteric kinase for one substrate. We let K be the kinase that exists in two conformations: K_r (relaxed state) and K_t (tensed state). Each of the conformations acts as a kinase for a common substrate S. We let S_p denote the phosphorylated form of the substrate. We assume that the intermediate kinase-substrate complexes, K_rS and K_tS, also undergo conformational change.

These considerations give rise to a reaction network with the following reactions:

- Phosphorylation of S:

$$K_r + S \underset{\kappa_2}{\overset{\kappa_1}{\rightleftharpoons}} K_rS \xrightarrow{\kappa_3} K_r + S_p \qquad\qquad K_t + S \underset{\kappa_5}{\overset{\kappa_4}{\rightleftharpoons}} K_tS \xrightarrow{\kappa_6} K_t + S_p$$

- Dephosphorylation of S_p:

$$S_p \xrightarrow{\kappa_7} S$$

- Conformational change:

$$K_r \underset{\kappa_9}{\overset{\kappa_8}{\rightleftharpoons}} K_t \qquad\qquad K_rS \underset{\kappa_{11}}{\overset{\kappa_{10}}{\rightleftharpoons}} K_tS.$$

We denote the concentration of the 6 species of the network as follows:

$$x_1 := [K_r] \quad x_2 := [K_t] \quad x_3 := [K_rS] \quad x_4 := [K_tS] \quad x_5 := [S] \quad x_6 := [S_p].$$

Under the law of mass action, the dynamics of the concentrations is modeled over time by the following system of ordinary differential equations:

$$\begin{aligned}
\dot{x}_1 &= -\kappa_1 x_1 x_5 + (\kappa_2 + \kappa_3) x_3 - \kappa_8 x_1 + \kappa_9 x_2\\
\dot{x}_2 &= -\kappa_4 x_2 x_5 + (\kappa_5 + \kappa_6) x_4 + \kappa_8 x_1 - \kappa_9 x_2\\
\dot{x}_3 &= \kappa_1 x_1 x_5 - (\kappa_2 + \kappa_3) x_3 - \kappa_{10} x_3 + \kappa_{11} x_4\\
\dot{x}_4 &= \kappa_4 x_2 x_5 - (\kappa_5 + \kappa_6) x_4 + \kappa_{10} x_3 - \kappa_{11} x_4\\
\dot{x}_5 &= -\kappa_1 x_1 x_5 - \kappa_4 x_2 x_5 + \kappa_2 x_3 + \kappa_5 x_4 + \kappa_7 x_6\\
\dot{x}_6 &= \kappa_3 x_3 + \kappa_6 x_4 - \kappa_7 x_6,
\end{aligned}$$

where $\dot{x}$ denotes the derivative of x with respect to time t and explicit reference to time t is omitted, that is, $x_* = x_*(t)$ and $\dot{x}_* = \dot{x}_*(t)$.

Since

$$\dot{x}_1 + \dot{x}_2 + \dot{x}_3 + \dot{x}_4 = 0 \quad \text{and} \quad \dot{x}_3 + \dot{x}_4 + \dot{x}_5 + \dot{x}_6 = 0,$$

the sums $x_1+x_2+x_3+x_4$ and $x_3+x_4+x_5+x_6$ are constant over time. These sums correspond to conservation of the total amounts of kinase and substrate, respectively. This leads to the following two *conservation laws*:

$$x_1 + x_2 + x_3 + x_4 = K_{\text{tot}} \quad \text{and} \quad x_3 + x_4 + x_5 + x_6 = S_{\text{tot}}. \tag{1}$$

Here $K_{\text{tot}}, S_{\text{tot}} > 0$ are positive *total amounts*.

We denote by $x = (x_1, \dots, x_6)$ the vector of concentrations and by $\kappa = (\kappa_1, \dots, \kappa_{11})$ the vector of reaction rate constants.

1.2 Summary of results

The results for the model with one allosteric kinase can be summarised in the following way. In subsection 1.4 we show that the steady states of the system can be given in terms of the concentration x_5 of the substrate S only. That is, for a given K_{tot} knowing the value of x_5 at steady state allows us to calculate the value of the remaining concentrations at steady state. Further, we show that the system can have up to three positive steady states by choosing the reaction rate constants and total amounts appropriately.

In subsections 1.5-1.7 we study necessary and sufficient conditions for multistationarity to occur. In subsection 1.5 necessary conditions for multistationarity on the reaction rate constants and the total amounts are given. Specifically, we find a necessary condition for multistationarity of the form

$$\alpha_1 K_{\text{tot}} + \alpha_2 < S_{\text{tot}} < \alpha_3 K_{\text{tot}} + \alpha_4, \tag{2}$$

where $\alpha_1, \ldots, \alpha_4$ depend on the reaction rate constants, see (10).

In subsection 1.6 we focus on conditions that are both necessary and sufficient for multistationarity. In subsection 1.6.1 we show that if the following inequality on the *reaction rate constants* is fulfilled, then the system is multistationary by choosing appropriate total amounts:

$$(\kappa_3 - \kappa_6)(\eta_r\kappa_9\kappa_{10} - \eta_t\kappa_8\kappa_{11}) > ((\kappa_6 + \kappa_7)\kappa_{10} + (\kappa_3 + \kappa_7)\kappa_{11})(\eta_r\kappa_{10} + \eta_t\kappa_{11})$$

where

$$\eta_r = \frac{\kappa_1}{\kappa_2 + \kappa_3} \qquad \text{and} \qquad \eta_t = \frac{\kappa_4}{\kappa_5 + \kappa_6}.$$

If the inequality is not fulfilled, then there cannot be multistationarity for any choice of total amounts. Moreover, by inspecting the inequality, other necessary conditions for multistationarity might be deduced. For example, one of the following two constraints is necessary for multistationarity to occur:

(a) $\kappa_3 > \kappa_6$ and $\eta_r\kappa_9\kappa_{10} > \eta_t\kappa_8\kappa_{11}$.

(b) $\kappa_3 < \kappa_6$ and $\eta_r\kappa_9\kappa_{10} < \eta_t\kappa_8\kappa_{11}$.

If a set of reaction rate constants fulfils the necessary and sufficient conditions for multistationarity, then the next question is to find total amounts for which it occurs. The linear inequalities in S_{tot} and K_{tot} given in (2) restrict the possible values considerably. However, it is also possible to give necessary and sufficient conditions involving all parameters, that is, the reaction rate constants *and* the total amounts. These conditions are given in equations (15) and (16) in subsection 1.6.2.

In subsection 1.7 we discuss how to explicitly find parameter sets for which multistationarity arises, using the conditions discussed above, and illustrate it with an example.

In subsection 1.8 we explore the relationship between the species concentrations at steady state and the concentration of the substrate S (x_5) at steady state. To this end we use the explicit descriptions of the species concentrations at steady state as functions of x_5, found in subsection 1.4. Furthermore we illustrate that the steady states of the system *cannot* be given in terms of the concentration x_6 of the modified substrate S_p alone. The reason is that x_5 cannot be expressed in terms of x_6 when multistationarity occurs. This occurs because the substrates S and S_p do *not* appear in a symmetric way in the reactions.

In subsection 1.9, we consider bifurcation plots in the multistationary setting. We study the effect of changing the total amounts of the substrate and the kinase on the number of steady states. We encounter here again the necessary and sufficient conditions from subsection 1.6.

1.3 Model reduction and multistationarity

In order to study the number of steady states of the networks we are interested in, we make use of a couple of results that allow us to simplify the network while keeping information on the number of steady states.

The first result concerns **removal of intermediates.** Intermediates are species in the network that do not appear interacting with any other species. For example the species K_rS and K_tS are intermediates.

Given a network, we can obtain a reduced network by "removing" some intermediates, one at a time. This is done in the following way. Say we want to remove an intermediate Y from the network. We remove all reactions of the original network that involve Y and add a reaction

$$y \to y' \qquad \text{whenever} \qquad y \to Y \to y' \quad \text{with} \quad y \neq y'$$

belongs to the original network. Here y and y' are the reactant of a reaction $y \to Y$ and product of a reaction $Y \to y'$, respectively.

To illustrate this, we consider the removal of the intermediate K_rS of the network in subsection 1.1. The reactions of the reduced network are obtained by considering all length 2 paths of the original network that go through K_rS. We have four such paths:

$$\begin{array}{cc} K_r + S \longrightarrow K_rS \longrightarrow K_r + S_p & \qquad K_r + S \longrightarrow K_rS \longrightarrow K_tS \\ K_tS \longrightarrow K_rS \longrightarrow K_r + S_p & \qquad K_tS \longrightarrow K_rS \longrightarrow K_r + S. \end{array}$$

By "collapsing" these paths and leaving the reactions that do not involve K_rS unaltered, we obtain the following reduced network:

$$\begin{array}{lll} K_r + S \longrightarrow K_r + S_p & \qquad K_tS \rightleftharpoons K_r + S & \qquad S_p \longrightarrow S \\ K_t + S \rightleftharpoons K_tS \longrightarrow K_t + S_p & \qquad K_tS \longrightarrow K_r + S_p & \qquad K_r \rightleftharpoons K_t. \end{array}$$

Clearly the process can be repeated now choosing another intermediate, for example K_tS. In this way we can obtain reduced networks by removing several intermediates.

Our interest in reduction by removal of intermediates is explained in the following theorem: if the reduced network admits N positive steady states, then so does the original network. This theorem and its proof can be found in [4].

Theorem 1 ([4])**.** *Consider a chemical reaction network G and let G' be the reduced network obtained by the removal of some intermediates as described above. If G' has N non-degenerate positive steady states for some choice of reaction rate constants and total amounts, then there is a choice of reaction rate constants and total amounts such that G has at least N corresponding non-degenerate positive steady states. Moreover, if all the steady states are hyperbolic, then the correspondence preserves the asymptotic stability of the steady states.*

The condition that the steady state is non-degenerate and hyperbolic is technical. A steady state is non-degenerate if the Jacobian map of the system when evaluated at the steady state and restricted to the *stoichiometric subspace* (defined by the conservation law

equations equated to zero), is non-singular. A hyperbolic steady state is a steady state for which the eigenvalues of the Jacobian of the system when evaluated at the steady state and restricted to the stoichiometric subspace have non-zero real part. These conditions are fulfilled in our applications of the theorem.

The other result we are interested in concerns **subnetworks**. A subnetwork of a network is obtained by removing some of the reactions of the original network. For example, by making some reversible reactions irreversible, we obtain a subnetwork. Observe that removal of a reaction corresponds to setting its reaction rate constant to zero in the ODE system. For example, making the reversible reaction $K_r + S \underset{\kappa_2}{\overset{\kappa_1}{\rightleftharpoons}} K_rS$ irreversible corresponds to letting $\kappa_2 = 0$ in the ODE system.

Under some conditions, knowing that a subnetwork admits N positive steady states is enough to conclude that this is also the case for the original network. A sufficient condition for this to hold is that the subnetwork and the network have the same species and conservation laws (or stated more precisely, the two networks have the same stoichiometric subspace). This theorem and its proof can be found in [6].

Theorem 2 ([6]). *Let G be a chemical reaction network and G' be a subnetwork of G such that G and G' have the same stoichiometric subspace. If there exist reaction rate constants and total amounts such that G' has N non-degenerate positive steady states, then there exist reaction rate constants and total amounts such that G has at least N non-degenerate positive steady states. Moreover, if all the steady states are hyperbolic, then the correspondence preserves the asymptotic stability of the steady states.*

The theorem can be applied to subnetworks obtained by making some reversible reactions irreversible, because the conservation laws, and thus the stoichiometric subspace, are not changed.

We will use these two reduction strategies in sections 2 and 3. In both sections we find the number of steady states of a reduced network, obtained either by removal of intermediates, reactions or both. These two results allow us then to deduce that the original network of interest has at least the same number of steady states (for some choice of reaction rate constants and total amounts).

Note that reduced networks might not be biologically realistic. In this text we use reduction as a mathematical simplification tool to obtain results for the networks of interest.

1.4 Positive steady states

1.4.1 The steady states as functions of [S]

The positive steady states of the system are the solutions to the equations $\dot{x}_1 = 0, \dots, \dot{x}_6 = 0$, constrained by the conservation laws (1). Due to the conservation laws, we can disregard two of the equations: we disregard the equations $\dot{x}_1 = 0$ and $\dot{x}_5 = 0$.

Consider first the system of equations given by $\dot{x}_2 = \dot{x}_3 = \dot{x}_4 = \dot{x}_6 = 0$ and the first

conservation law in (1). That is, consider the system of equations:

$$\begin{aligned}
0 &= -\kappa_4 x_2 x_5 + (\kappa_5+\kappa_6)x_4 + \kappa_8 x_1 - \kappa_9 x_2\\
0 &= \kappa_1 x_1 x_5 - (\kappa_2+\kappa_3)x_3 - \kappa_{10}x_3 + \kappa_{11}x_4\\
0 &= \kappa_4 x_2 x_5 - (\kappa_5+\kappa_6)x_4 + \kappa_{10}x_3 - \kappa_{11}x_4\\
0 &= \kappa_3 x_3 + \kappa_6 x_4 - \kappa_7 x_6,\\
K_{\text{tot}} &= x_1 + x_2 + x_3 + x_4.
\end{aligned} \tag{3}$$

This is a nonlinear polynomial system in the variables $x_1,\dots,x_6$ with coefficients involving the reaction rate constants. However, it is a linear system when it is considered only in the variables x_1,x_2,x_3,x_4,x_6 and x_5 is treated as an additional parameter of the system and hence part of the coefficients. This linear system can be solved using a symbolic software such as Mathematica or Maple, obtaining the following algebraic expressions for x_1,x_2,x_3,x_4,x_6 at steady state, which depend on the value of x_5 at steady state:

$$x_1 = \frac{K_{\text{tot}}}{q(x)}\Big((\kappa_2+\kappa_3)\kappa_4\kappa_{11}x_5 + \kappa_9((\kappa_2+\kappa_3)(\kappa_5+\kappa_6) + (\kappa_2+\kappa_3)\kappa_{11} + (\kappa_5+\kappa_6)\kappa_{10})\Big) \tag{4}$$

$$x_2 = \frac{K_{\text{tot}}}{q(x)}\Big((\kappa_5+\kappa_6)\kappa_1\kappa_{10}x_5 + \kappa_8((\kappa_2+\kappa_3)(\kappa_5+\kappa_6) + (\kappa_2+\kappa_3)\kappa_{11} + (\kappa_5+\kappa_6)\kappa_{10})\Big) \tag{5}$$

$$x_3 = \frac{K_{\text{tot}}x_5}{q(x)}\Big(\kappa_1\kappa_4\kappa_{11}x_5 + \kappa_1\kappa_9(\kappa_5+\kappa_6+\kappa_{11}) + \kappa_4\kappa_8\kappa_{11}\Big) \tag{6}$$

$$x_4 = \frac{K_{\text{tot}}x_5}{q(x)}\Big(\kappa_1\kappa_4\kappa_{10}x_5 + \kappa_4\kappa_8(\kappa_2+\kappa_3+\kappa_{10}) + \kappa_1\kappa_9\kappa_{10}\Big) \tag{7}$$

$$\begin{aligned}
x_6 = \frac{K_{\text{tot}}x_5}{\kappa_7 q(x)}\Big(&\kappa_1\kappa_4\left(\kappa_3\kappa_{11}+\kappa_6\kappa_{10}\right)x_5 + \kappa_1\kappa_3\kappa_9(\kappa_5+\kappa_{11}) + \kappa_4\kappa_8(\kappa_2\kappa_6+\kappa_3\kappa_{11}) +\\
&\kappa_6(\kappa_3+\kappa_{10})(\kappa_1\kappa_9+\kappa_4\kappa_8)\Big),
\end{aligned} \tag{8}$$

where $q(x)$ is the following polynomial in x_5:

$$\begin{aligned}
q(x) :=\,&\kappa_1\kappa_4(\kappa_{10}+\kappa_{11})x_5^2\\
&+ ((\kappa_2+\kappa_3)\kappa_4(\kappa_8+\kappa_{11}) + (\kappa_5+\kappa_6)\kappa_1(\kappa_9+\kappa_{10}) + (\kappa_{10}+\kappa_{11})(\kappa_1\kappa_9+\kappa_4\kappa_8))x_5\\
&+ (\kappa_8+\kappa_9)((\kappa_2+\kappa_3)(\kappa_5+\kappa_6+\kappa_{11}) + \kappa_{10}(\kappa_5+\kappa_6)).
\end{aligned}$$

The expressions for x_1,x_2,x_3,x_4,x_6 are positive provided x_5 is positive.

1.4.2 The steady state polynomial

All concentrations are expressed as functions of x_5. After replacing x_3,x_4,x_6 in the second conservation law in (1) by their expressions in (6),(7),(8), we obtain that the value of x_5 at a positive steady state satisfies the equation:

$$\begin{aligned}
0 = (x_5 - S_{\text{tot}}) + \frac{K_{\text{tot}}x_5}{\kappa_7 q(x)}\Big(&\big(\kappa_1\kappa_4\kappa_{11}x_5 + \kappa_1\kappa_9(\kappa_5+\kappa_6+\kappa_{11}) + \kappa_4\kappa_8\kappa_{11}\big)\kappa_7\\
&+ \big(\kappa_1\kappa_4\kappa_{10}x_5 + \kappa_4\kappa_8(\kappa_2+\kappa_3+\kappa_{10}) + \kappa_1\kappa_9\kappa_{10}\big)\kappa_7\\
&+ \big(\kappa_1\kappa_4\left(\kappa_3\kappa_{11}+\kappa_6\kappa_{10}\right)x_5 + \kappa_1\kappa_3\kappa_9(\kappa_5+\kappa_{11}) + \kappa_4\kappa_8(\kappa_2\kappa_6+\kappa_3\kappa_{11})\\
&+ \kappa_6(\kappa_3+\kappa_{10})(\kappa_1\kappa_9+\kappa_4\kappa_8)\big)\Big).
\end{aligned}$$

By multiplying this equation by the denominator $\kappa_7 q(x)$, the positive solutions to the above equation agree with the positive solutions to the following polynomial in x_5:

$$
\begin{aligned}
p(x_5) =& \kappa_1\kappa_4\kappa_7(\kappa_{10}+\kappa_{11})x_5^3 \qquad (9)\\
&+\Big(K_{\text{tot}}\kappa_1\kappa_4\big((\kappa_6+\kappa_7)\kappa_{10}+(\kappa_3+\kappa_7)\kappa_{11}\big)-S_{\text{tot}}\kappa_1\kappa_4\kappa_7(\kappa_{10}+\kappa_{11})\\
&+(\kappa_2+\kappa_3)\kappa_4\kappa_7(\kappa_8+\kappa_{11})+(\kappa_5+\kappa_6)\kappa_1\kappa_7(\kappa_9+\kappa_{10})+\kappa_7(\kappa_1\kappa_9+\kappa_4\kappa_8)(\kappa_{10}+\kappa_{11})\Big)x_5^2\\
&+\Big((\kappa_1\kappa_9+\kappa_4\kappa_8)\big(K_{\text{tot}}((\kappa_6+\kappa_7)\kappa_{10}+(\kappa_3+\kappa_7)\kappa_{11})-S_{\text{tot}}\kappa_7(\kappa_{10}+\kappa_{11})\big)\\
&+(\kappa_2+\kappa_3)\kappa_4\big(K_{\text{tot}}\kappa_8(\kappa_6+\kappa_7)-S_{\text{tot}}\kappa_7(\kappa_8+\kappa_{11})\big)\\
&+(\kappa_5+\kappa_6)\kappa_1\big(K_{\text{tot}}\kappa_9(\kappa_3+\kappa_7)-S_{\text{tot}}\kappa_7(\kappa_9+\kappa_{10})\big)\\
&+\big((\kappa_2+\kappa_3)\kappa_{11}+(\kappa_5+\kappa_6)\kappa_{10}+(\kappa_2+\kappa_3)(\kappa_5+\kappa_6)\big)\kappa_7(\kappa_8+\kappa_9)\Big)x_5\\
&-S_{\text{tot}}\kappa_7(\kappa_8+\kappa_9)\big((\kappa_2+\kappa_3)(\kappa_5+\kappa_6)+(\kappa_2+\kappa_3)\kappa_{11}+(\kappa_5+\kappa_6)\kappa_{10}\big).
\end{aligned}
$$

The polynomial $p(x_5)$ has degree 3. Any positive root of this polynomial gives rise to a positive steady state using the expressions (4)-(8) and, similarly, the value of x_5 for any positive steady state of the system is a root of the polynomial. That is, positive steady states of the network fulfilling the conservation laws (1) are in one-to-one correspondence with the positive roots of this polynomial.

We note that this polynomial has at least one positive root since $p(0) < 0$ and $p(+\infty) > 0$. Moreover, the reaction rate constants and the total amounts can be chosen such that $p(x_5)$ has indeed three positive roots. Therefore, there exist reaction rate constants and total amounts such that the system has three positive steady states. This is first shown by giving a set of parameters such that the system has three positive steady states in subsection 1.8.

A formal mathematical proof of this result is given in subsection 2.4 where we study a generalisation of this model. The proof is based on showing that the result holds for a reduced model and then using Theorem 2 (Section 1.3).

1.5 Necessary conditions for multistationarity

In this section we use the Descartes' rule of signs on the number of positive roots of a polynomial. The rule says that the number of positive roots of a polynomial is smaller than or equal to the number of sign changes in the sequence of signs of the coefficients of the polynomial (ignoring the coefficients that are zero).

Proposition 3. *A* ***necessary condition*** *for the existence of three positive steady states is that*

$$
\begin{aligned}
&K_{\text{tot}}\kappa_1\kappa_4\big((\kappa_6+\kappa_7)\kappa_{10}+(\kappa_3+\kappa_7)\kappa_{11}\big)+(\kappa_2+\kappa_3)\kappa_4\kappa_7(\kappa_8+\kappa_{11})\\
&\quad+(\kappa_5+\kappa_6)\kappa_1\kappa_7(\kappa_9+\kappa_{10})+\kappa_7(\kappa_1\kappa_9+\kappa_4\kappa_8)(\kappa_{10}+\kappa_{11}) < S_{\text{tot}}\kappa_1\kappa_4\kappa_7(\kappa_{10}+\kappa_{11})
\end{aligned}
$$

and

$$
\begin{aligned}
&K_{\text{tot}}\Big((\kappa_1\kappa_9+\kappa_4\kappa_8)\big((\kappa_6+\kappa_7)\kappa_{10}+(\kappa_3+\kappa_7)\kappa_{11}\big)+(\kappa_2+\kappa_3)\kappa_4\kappa_8(\kappa_6+\kappa_7)\\
&+(\kappa_5+\kappa_6)\kappa_1\kappa_9(\kappa_3+\kappa_7)\Big)+\big((\kappa_2+\kappa_3)\kappa_{11}+(\kappa_5+\kappa_6)\kappa_{10}+(\kappa_2+\kappa_3)(\kappa_5+\kappa_6)\big)\kappa_7(\kappa_8+\kappa_9)\\
&\qquad > S_{\text{tot}}\kappa_7\Big((\kappa_1\kappa_9+\kappa_4\kappa_8)(\kappa_{10}+\kappa_{11})+(\kappa_2+\kappa_3)\kappa_4(\kappa_8+\kappa_{11})+(\kappa_5+\kappa_6)\kappa_1(\kappa_9+\kappa_{10})\Big).
\end{aligned}
$$

Proof. Following Descartes' rule of signs, a necessary condition for $p(x_5)$ to have three positive roots is that the coefficients of the polynomial have alternating signs, that is, there must be three sign changes in the sequence of signs of the coefficients of the polynomial and none of these coefficients can be zero. Since the leading coefficient of $p(x_5)$ is positive and the independent term is negative, a necessary condition is that the coefficient of degree 2 is negative (this gives the first condition in the statement) and the coefficient of degree 1 is positive (this gives the second condition in the statement). □

In contrast to the condition that will be derived in the next subsection, the two conditions of the proposition involve the total amounts. By isolating S_{tot} from both inequalities, these conditions can be rewritten as

$$\alpha_1 K_{\text{tot}} + \alpha_2 < S_{\text{tot}} < \alpha_3 K_{\text{tot}} + \alpha_4, \tag{10}$$

where:

$$\alpha_1 = \frac{\kappa_1\kappa_4\big((\kappa_6+\kappa_7)\kappa_{10} + (\kappa_3+\kappa_7)\kappa_{11}\big)}{\kappa_1\kappa_4\kappa_7(\kappa_{10}+\kappa_{11})},$$

$$\alpha_2 = \frac{(\kappa_2+\kappa_3)\kappa_4\kappa_7(\kappa_8+\kappa_{11}) + (\kappa_5+\kappa_6)\kappa_1\kappa_7(\kappa_9+\kappa_{10}) + \kappa_7(\kappa_1\kappa_9+\kappa_4\kappa_8)(\kappa_{10}+\kappa_{11})}{\kappa_1\kappa_4\kappa_7(\kappa_{10}+\kappa_{11})},$$

$$\alpha_3 = \frac{(\kappa_1\kappa_9+\kappa_4\kappa_8)((\kappa_6+\kappa_7)\kappa_{10}+(\kappa_3+\kappa_7)\kappa_{11}) + (\kappa_2+\kappa_3)\kappa_4\kappa_8(\kappa_6+\kappa_7) + (\kappa_5+\kappa_6)\kappa_1\kappa_9(\kappa_3+\kappa_7)}{\kappa_7\big((\kappa_1\kappa_9+\kappa_4\kappa_8)(\kappa_{10}+\kappa_{11}) + (\kappa_2+\kappa_3)\kappa_4(\kappa_8+\kappa_{11}) + (\kappa_5+\kappa_6)\kappa_1(\kappa_9+\kappa_{10})\big)},$$

$$\alpha_4 = \frac{\big((\kappa_2+\kappa_3)\kappa_{11} + (\kappa_5+\kappa_6)\kappa_{10} + (\kappa_2+\kappa_3)(\kappa_5+\kappa_6)\big)\kappa_7(\kappa_8+\kappa_9)}{\kappa_7\big((\kappa_1\kappa_9+\kappa_4\kappa_8)(\kappa_{10}+\kappa_{11}) + (\kappa_2+\kappa_3)\kappa_4(\kappa_8+\kappa_{11}) + (\kappa_5+\kappa_6)\kappa_1(\kappa_9+\kappa_{10})\big)}.$$

For each fixed value of K_{tot}, the solution to the system of inequalities (10) is either empty or an interval. Since $\alpha_i > 0$ for $i = 1, 2, 3, 4$, $\alpha_1 K_{\text{tot}} + \alpha_2$ and $\alpha_3 K_{\text{tot}} + \alpha_4$ are increasing straight lines in K_{tot} with positive intercept. Therefore the region is described by a sector intersected with the positive orthant of $\mathbb{R}^2$. If the two lines are parallel, then the valid region is the region between the two lines intersected with the positive orthant.

An example of how such a sector might look like is given in Example 7 below.

1.6 Necessary and sufficient conditions for multistationarity

1.6.1 Conditions involving only reaction rate constants

In order to find sufficient conditions for multistationarity involving only reaction rate constants, we apply the strategy introduced in [1]. The strategy is based on Brouwer Degree Theory. In [2], sufficient conditions for multistationarity, based on the reaction rate constants only, were found for a two-site phosphorylation cycle in which both the kinase and the phosphatase follow a sequential and distributive mechanism.

Proposition 4. *For a given choice of reaction rate constants κ, there exist total amounts K_{tot} and S_{tot} such that the network admits multiple positive steady states if and only if*

$$(\kappa_3 - \kappa_6)(\eta_r\kappa_9\kappa_{10} - \eta_t\kappa_8\kappa_{11}) > ((\kappa_6+\kappa_7)\kappa_{10} + (\kappa_3+\kappa_7)\kappa_{11})(\eta_r\kappa_{10} + \eta_t\kappa_{11}) \tag{11}$$

where

$$\eta_r = \frac{\kappa_1}{\kappa_2+\kappa_3} \quad \textit{and} \quad \eta_t = \frac{\kappa_4}{\kappa_5+\kappa_6}$$

are the inverses of the Michaelis-Menten constants for the different allosteric states of the kinase, i.e. for K_r and K_t respectively.

Equivalently, the network admits multiple positive steady states if and only if

$$\alpha(\kappa) = \kappa_1\kappa_4(\kappa_3-\kappa_6)\big(\kappa_1(\kappa_5+\kappa_6)\kappa_9\kappa_{10} - \kappa_4(\kappa_2+\kappa_3)\kappa_8\kappa_{11}\big) \\ - \kappa_1\kappa_4\big((\kappa_6+\kappa_7)\kappa_{10}+(\kappa_3+\kappa_7)\kappa_{11}\big)\big(\kappa_1(\kappa_5+\kappa_6)\kappa_{10}+\kappa_4\kappa_{11}(\kappa_2+\kappa_3)\big). \tag{12}$$

is positive.

Proof. We apply the strategy from [1]. The steps of the procedure are as follows:

(i) Consider the function $f_\kappa(x)$ given by the two conservation laws and the expressions for $\dot{x_2}, \dot{x_3}, \dot{x_4}$ and $\dot{x_6}$, that is, the right-hand side of the expressions in (3) together with the second equation in (1):

$$f_\kappa(x) = (-\kappa_4x_2x_5+(\kappa_5+\kappa_6)x_4+\kappa_8x_1-\kappa_9x_2, \kappa_1x_1x_5-(\kappa_2+\kappa_3)x_3-\kappa_{10}x_3+\kappa_{11}x_4, \\ \kappa_4x_2x_5-(\kappa_5+\kappa_6)x_4+\kappa_{10}x_3-\kappa_{11}x_4, \kappa_3x_3+\kappa_6x_4-\kappa_7x_6, \\ x_1+x_2+x_3+x_4, x_3+x_4+x_5+x_6).$$

Compute the Jacobian matrix of $f_\kappa(x)$:

$$J_\kappa(x) = \begin{pmatrix} \kappa_8 & -\kappa_4x_5-\kappa_9 & 0 & \kappa_5+\kappa_6 & -\kappa_4x_2 & 0 \\ \kappa_1x_5 & 0 & -\kappa_2-\kappa_3-\kappa_{10} & \kappa_{11} & \kappa_1x_1 & 0 \\ 0 & \kappa_4x_5 & \kappa_{10} & -\kappa_5-\kappa_6-\kappa_{11} & \kappa_4x_2 & 0 \\ 0 & 0 & \kappa_3 & \kappa_6 & 0 & -\kappa_7 \\ 1 & 1 & 1 & 1 & 0 & 0 \\ 0 & 0 & 1 & 1 & 1 & 1 \end{pmatrix} \tag{13}$$

and its determinant, $\det(J_\kappa(x))$.

(ii) Express x_2, x_3, x_4 and x_6 at steady state in terms of x_1 and x_5. That is, consider the steady state equations $\dot{x}_2=\dot{x}_3=\dot{x}_4=\dot{x}_6=0$ and solve them for x_2, x_3, x_4, x_6 in terms of x_1, x_5. This gives the following expression:

$$\begin{aligned} x_2 &= \frac{(\kappa_5+\kappa_6)\kappa_1\kappa_{10}x_1x_5+\kappa_8\big((\kappa_5+\kappa_6+\kappa_{11})(\kappa_2+\kappa_3)+\kappa_{10}(\kappa_5+\kappa_6)\big)x_1}{\chi_\kappa(x_5)} \\ x_3 &= \frac{\kappa_1\kappa_4\kappa_{11}x_1x_5^2+\big(\kappa_1\kappa_9(\kappa_5+\kappa_6+\kappa_{11})+\kappa_4\kappa_8\kappa_{11}\big)x_1x_5}{\chi_\kappa(x_5)} \\ x_4 &= \frac{\kappa_1\kappa_4\kappa_{10}x_1x_5^2+\big(\kappa_1\kappa_9\kappa_{10}+\kappa_4\kappa_8(\kappa_2+\kappa_3+\kappa_{10})\big)x_1x_5}{\chi_\kappa(x_5)} \\ x_6 &= \frac{\kappa_1\kappa_4(\kappa_3\kappa_{11}+\kappa_6\kappa_{10})x_1x_5^2+\kappa_1\kappa_9\big(\kappa_3(\kappa_5+\kappa_6+\kappa_{11})+\kappa_6\kappa_{10}\big)x_1x_5}{\chi_\kappa(x_5)} \\ &\quad + \frac{\kappa_4\kappa_8\big(\kappa_6(\kappa_2+\kappa_3+\kappa_{10})+\kappa_3\kappa_{11}\big)x_1x_5}{\chi_\kappa(x_5)}, \end{aligned} \tag{14}$$

where

$$\chi_\kappa(x_5) = \kappa_4\kappa_{11}(\kappa_2+\kappa_3)x_5+\kappa_9(\kappa_2+\kappa_3)(\kappa_5+\kappa_6+\kappa_{11})+(\kappa_5+\kappa_6)\kappa_9\kappa_{10}.$$

(iii) Substitute the values of x_2, x_3, x_4, x_6 from (14) into the determinant of the Jacobian matrix in (13). The resulting expression is the quotient of a polynomial $b_\kappa(x_1, x_5)$ in x_1, x_5 and the polynomial $\chi_\kappa(x_5)$. Note that all coefficients of the denominator are positive.

Based on Brouwer Degree Theory (see [1]), we conclude that multistationarity occurs if and only if the polynomial $b_\kappa(x_1,x_5)$ is positive for some positive values of x_1,x_5. Specifically, for a given choice of reaction rate constants κ, there exist total amounts such that the system displays multistationarity if and only if $b_\kappa(x_1,x_5)$ is positive for some positive values of x_1,x_5.

Viewed as a polynomial in x_1,x_5, $b_\kappa(x_1,x_5)$ is a polynomial in the monomials 1, x_1, x_5, x_1x_5, x_5^2, $x_1x_5^2$, x_5^3. Further all coefficients of $b_\kappa(x_1,x_5)$ are polynomials in κ. All coefficients have negative sign, independently of the values of κ_i, except for the coefficient of $x_1x_5^2$ which is $\alpha(\kappa)$. Clearly, if this coefficient is also negative, then *all* coefficients of the polynomial are negative and thus $b_\kappa(x_1,x_5)$ is negative for all positive values of x_1,x_5. As a consequence multistationarity cannot occur for this choice of reaction rate constants κ.

Assume now that $\alpha(\kappa)$ is positive. We show that in this case the polynomial $b_\kappa(x_1,x_5)$ is positive for some values of x_1,x_5. Let $x_5=T$ and $x_1=T^2$. Then $b_\kappa(T^2,T)$ is a polynomial in T of degree 4 with leading positive coefficient $\alpha(\kappa)$. By letting T be arbitrarily large, $b_\kappa(T^2,T)$ becomes eventually positive. We conclude that $b_\kappa(x_1,x_5)$ is positive for some values of x_1,x_5 and hence that there exist total amounts such that the network with reaction rate constants κ has more than one positive steady state.

This shows that multistationarity occurs if and only if $\alpha(\kappa)$ is positive. After rearranging the terms of the coefficient $\alpha(\kappa)$, the inequality $\alpha(\kappa)>0$ is equivalent to the inequality given in the statement of the proposition, i.e. inequality (11). □

By inspecting inequality (11), we can find some simple necessary conditions for multistationarity. For example:

- Either $\kappa_9\kappa_{10}\neq 0$ or $\kappa_8\kappa_{11}\neq 0$ (or both are non-zero). That is, allosteric changes must occur both for the kinase and the kinase-substrate complexes.
- Since the left-hand side of the inequality must be positive for the inequality to hold, we deduce that one of the following conditions is necessary for multistationarity:
  (a) $\kappa_3>\kappa_6$ and $\eta_r\kappa_9\kappa_{10}>\eta_t\kappa_8\kappa_{11}$.
  (b) $\kappa_3<\kappa_6$ and $\eta_r\kappa_9\kappa_{10}<\eta_t\kappa_8\kappa_{11}$.

1.6.2 Conditions involving reaction rate constants and total amounts

Here we provide necessary and sufficient conditions on *all* parameters (reaction rate constants and total amounts) of the system for multistationarity to occur. To obtain the conditions, we apply Sturm's Theorem:

Theorem 5 (Sturm). *Let $p(x)$ be a real polynomial. Define recursively the Sturm sequence by*

$$p_0(x)=p(x),\ p_1(x)=p'(x),\quad \textit{and}\quad p_{i+1}(x)=-\mathrm{rem}(p_{i-1},p_i),$$

for $i\geq 1$, where $\mathrm{rem}(p_{i-1},p_i)$ denotes the reminder of p_{i-1} divided by p_i. The sequence stops when $p_{i+1}=0$. Let p_m be the last nonzero polynomial.

For $c\in\mathbb{R}$, let $\sigma(c)$ be the number of sign changes in the sequence $p_0(c),\dots,p_m(c)$. Let $a<b$ and assume that neither a nor b are multiple roots of $p(x)$. Then $\sigma(a)-\sigma(b)$ is the number of distinct roots of $p(x)$ in the interval $(a,b]$.

Proposition 6. *Given reaction rate constants κ and total amounts K_{tot} and S_{tot}, the network has three positive steady states if and only if the following inequalities are satisfied*

$$\begin{aligned} a_2 &> 0 \\ -9a_0a_3 + a_1a_2 &< 0 \\ 27a_0^2a_3^2 - 18a_0a_1a_2a_3 + 4a_0a_2^3 + 4a_1^3a_3 - a_1^2a_2^2 &< 0 \\ -6a_0a_2 + 2a_1^2 &> 0, \end{aligned} \tag{15}$$

where

$$\begin{aligned} a_0 =\ & \kappa_1\kappa_4\kappa_7(\kappa_{10} + \kappa_{11}) \\ a_1 =\ & K_{\text{tot}}\kappa_1\kappa_4((\kappa_6 + \kappa_7)\kappa_{10} + (\kappa_3 + \kappa_7)\kappa_{11}) - S_{\text{tot}}\kappa_1\kappa_4\kappa_7(\kappa_{10} + \kappa_{11}) \\ & + (\kappa_2 + \kappa_3)\kappa_4\kappa_7(\kappa_8 + \kappa_{11}) + (\kappa_5 + \kappa_6)\kappa_1\kappa_7(\kappa_9 + \kappa_{10}) + \kappa_7(\kappa_1\kappa_9 + \kappa_4\kappa_8)(\kappa_{10} + \kappa_{11}) \\ a_2 =\ & (\kappa_1\kappa_9 + \kappa_4\kappa_8)(K_{\text{tot}}((\kappa_6 + \kappa_7)\kappa_{10} + (\kappa_3 + \kappa_7)\kappa_{11}) - S_{\text{tot}}\kappa_7(\kappa_{10} + \kappa_{11})) \\ & + (\kappa_2 + \kappa_3)\kappa_4(K_{\text{tot}}\kappa_8(\kappa_6 + \kappa_7) - S_{\text{tot}}\kappa_7(\kappa_8 + \kappa_{11})) \\ & + (\kappa_5 + \kappa_6)\kappa_1(K_{\text{tot}}\kappa_9(\kappa_3 + \kappa_7) - S_{\text{tot}}\kappa_7(\kappa_9 + \kappa_{10})) \\ & + ((\kappa_2 + \kappa_3)\kappa_{11} + (\kappa_5 + \kappa_6)\kappa_{10} + (\kappa_2 + \kappa_3)(\kappa_5 + \kappa_6))\kappa_7(\kappa_8 + \kappa_9) \\ a_3 =\ & - S_{\text{tot}}\kappa_7(\kappa_8 + \kappa_9)((\kappa_2 + \kappa_3)(\kappa_5 + \kappa_6) + (\kappa_2 + \kappa_3)\kappa_{11} + (\kappa_5 + \kappa_6)\kappa_{10}). \end{aligned} \tag{16}$$

Proof. We are interested in the positive roots of the polynomial $p(x) = p(x_5)$ in (9). In order to apply Sturm's theorem, we should take $a = 0$ and b so large that all positive roots are in $(a, b] = (0, b]$. If b is chosen arbitrarily large, then the signs of $p_0(b), \dots, p_m(b)$ are determined by the leading coefficients of the polynomials $p_0, \dots, p_m$. Because b is an arbitrarily large number, we write $b = +\infty$ and the sequence is written as $p_0(+\infty), \dots, p_m(+\infty)$. Observe that $a = 0$ is not a root of $p(x)$ and hence the hypothesis of Sturm's theorem applies.

According to the theorem, $\sigma(0) - \sigma(+\infty)$ equals the number of distinct positive roots of $p(x)$. In our case, we have $m = 3$, that is, $p_4(x) = 0$ (see below), and hence $0 \leq \sigma(c) \leq 3$ for $c \geq 0$. Therefore, the number of distinct roots will be 3, that is, there will be three positive steady states, if and only if $\sigma(0) = 3$ and $\sigma(+\infty) = 0$.

We computed in Maple the Sturm sequence $p_0(x), \dots, p_3(x)$ ($p_4(x) = 0$). For a generic polynomial of degree 3, $p_0(x) = a_0x^3 + a_1x^2 + a_2x + a_3$, the sequence is:

$$\begin{aligned} p_0(x) &= a_0x^3 + a_1x^2 + a_2x + a_3 \\ p_1(x) &= 3a_0x^2 + 2a_1x + a_2 \\ p_2(x) &= -\frac{6a_0a_2x - 2a_1^2x + 9a_0a_3 - a_1a_2}{9a_0} \\ p_3(x) &= -\frac{9a_0(27a_0^2a_3^2 - 18\, a_0a_1a_2a_3 + 4a_0a_2^3 + 4a_1^3a_3 - a_1^2a_2^2)}{4(3a_0a_2 - a_1^2)^2}. \end{aligned}$$

In our case, the coefficients are given by equation (16) in the statement of the proposition. Since $p_0(0) = a_3 < 0$, for $\sigma(0) = 3$ we need

$$p_1(0) > 0,\ p_2(0) < 0 \quad \text{and} \quad p_3(0) > 0.$$

On the other hand,

$$p_0(+\infty) = a_0 > 0 \quad \text{and} \quad p_1(+\infty) = 3a_0 > 0.$$

Therefore, for $\sigma(+\infty)=0$ we require $p_2(+\infty), p_3(+\infty)>0$.

The polynomial $p_3(x)$ has degree zero, and hence $p_3(0)=p_3(+\infty)$. Therefore, we are left with 4 conditions on the parameters that fully characterise the region of the parameter space with three positive steady states, namely

$$p_1(0)>0, \quad p_3(0)>0, \quad p_2(+\infty)>0 \quad \text{and} \quad p_2(0)<0.$$

Using that $a_0>0$ and $a_3<0$, these conditions simplify to the following conditions:

$$\begin{aligned} a_2 &> 0 && (p_1(0)>0)\\ -9a_0a_3+a_1a_2 &< 0 && (p_2(0)<0)\\ 27a_0^2a_3^2-18a_0a_1a_2a_3+4a_0a_2^3+4a_1^3a_3-a_1^2a_2^2 &< 0 && (p_3(0)>0)\\ -6a_0a_2+2a_1^2 &> 0 && (p_2(+\infty)>0). \end{aligned}$$

□

1.7 Necessary and sufficient conditions in practice

In order to find explicit values of the parameters such that the system exhibits multistationarity, the procedure is the following:

1. First, use the necessary and sufficient condition given in (11) to find appropriate values for the reaction rate constants.
2. Second, substitute these values of the reaction rate constants into (15). This yields a system of 4 inequalities in K_{tot} and S_{tot}. The positive values of $(K_{\text{tot}}, S_{\text{tot}})$ fulfilling the inequalities correspond to parameter sets for which there are three positive steady states. By the results above, there are always values of $(K_{\text{tot}}, S_{\text{tot}})$ for which this is the case.

After the first step we might use the necessary conditions for multistationarity from subsection 1.5, that is, using the inequalities in (10) instead of the conditions in (15). This gives (simpler) regions of the parameter space of total amounts containing all pairs $(K_{\text{tot}}, S_{\text{tot}})$ for which there is multistationarity. Remember though that not all pairs $(K_{\text{tot}}, S_{\text{tot}})$ satisfying the inequalities in (10) yield multistationarity as the conditions are only necessary and not sufficient.

Example 7. Consider the set of parameters

$$\begin{aligned} &\kappa_1=5, && \kappa_2=0.1, && \kappa_3=1, && \kappa_4=2, && \kappa_5=0.1, && \kappa_6=2,\\ &\kappa_7=0.01, && \kappa_8=0.8, && \kappa_9=0.1, && \kappa_{10}=0.01, && \kappa_{11}=0.1 \end{aligned} \tag{17}$$

for which (11) is satisfied. The system of inequalities (10) is

$$110.1K_{\text{tot}}+3.06<S_{\text{tot}}<144.16K_{\text{tot}}+0.65,$$

and the pairs $(K_{\text{tot}}, S_{\text{tot}})$ fulfilling the inequalities are highlighted in light blue in Figure 1(a,b).

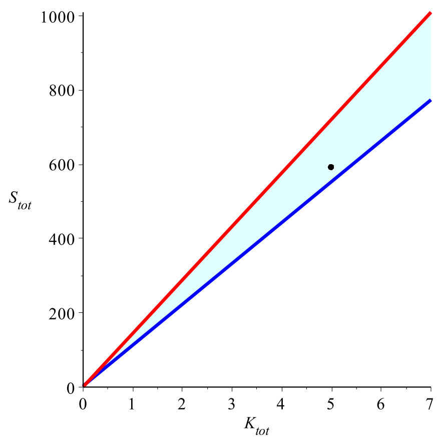

Figure 1a.

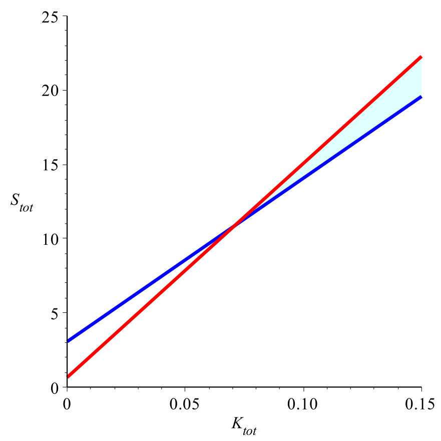

Figure 1b.

The plot in Figure 1b illustrates that for very small values of K_{tot} there is no value of S_{tot} for which the inequalities are satisfied. The dot in Figure 1a corresponds to $S_{\text{tot}} = 591$ and $K_{\text{tot}} = 5$, for which there is multistationarity.

We use the set of inequalities in (15) to find a precise characterization of the pairs of total amounts for which multistationarity occurs. The conditions translate into the following set of inequalities:

$$
\begin{aligned}
4.852K_{\text{tot}} - 0.03366S_{\text{tot}} + 0.02197 &> 0 \\
5.876K_{\text{tot}}^2 - 0.09414K_{\text{tot}}S_{\text{tot}} + 0.0003703S_{\text{tot}}^2 + 0.0008003S_{\text{tot}} + 0.1899K_{\text{tot}} + 0.0007395 &< 0 \\
-2.013\cdot 10^{-8}S_{\text{tot}}^4 - 34.531K_{\text{tot}}^4 + 0.9503S_{\text{tot}}K_{\text{tot}}^3 - 0.008961S_{\text{tot}}^2K_{\text{tot}}^2 + 0.00003108K_{\text{tot}}S_{\text{tot}}^3 & \\
-1.232\cdot 10^{-7}S_{\text{tot}}^3 + 2.795K_{\text{tot}}^3 - 0.04015S_{\text{tot}}K_{\text{tot}}^2 + 0.0001532K_{\text{tot}}S_{\text{tot}}^2 & \\
+0.02351K_{\text{tot}}^2 + 1.786\cdot 10^{-4}K_{\text{tot}}S_{\text{tot}} - 2.689\cdot 10^{-7}S_{\text{tot}}^2 & \\
+2.823\cdot 10^{-5}K_{\text{tot}} - 2.460\cdot 10^{-7}S_{\text{tot}} - 8.029\cdot 10^{-8} &< 0 \\
2.933K_{\text{tot}}^2 - 0.05328K_{\text{tot}}S_{\text{tot}} + 0.000242S_{\text{tot}}^2 - 0.1572K_{\text{tot}} + 0.0007405S_{\text{tot}} + 0.0008160 &> 0.
\end{aligned}
$$

One set of total amounts fulfilling the above system of inequalities is $S_{\text{tot}} = 591$ and $K_{\text{tot}} = 5$ (the point plotted in the figure above).

In the following two plots (Figure 2(a,b)) the yellow region is the region of common solutions to the first, second and fourth inequalities, and the blue region the solution to the third inequality. The intersection of the two regions is the solution set to the four inequalities. It is the small blue region inside the yellow region. Figure 2b is a magnification of Figure 2a, in which also the point $(5, 591)$ is shown.

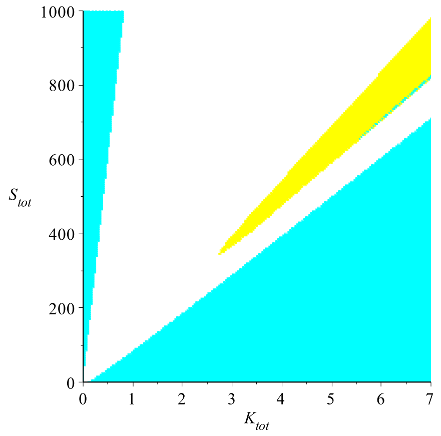

Figure 2a.

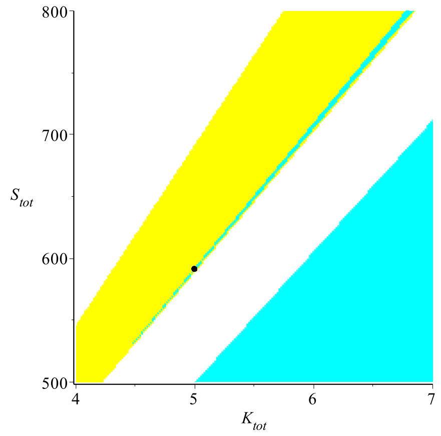

Figure 2b.

The region for which multistationarity exists is much smaller than the region given in Figure 1(a,b). Note that this example illustrates the region for S_{tot} and K_{tot} leading to multistationarity only for the specific set of parameters given in (17).

In practice, it is not straightforward to solve the Sturm's inequalities for S_{tot} and K_{tot}.

1.8 Describing the steady states

In this section we describe the intersection of the solution set to the steady state equations, with the linear space defined by $x_1 + x_2 + x_3 + x_4 = K_{\text{tot}}$, using the expressions (4)-(8). To illustrate the results of this section we choose the set of parameters for which multistationarity occurs given in (17):

$$\begin{array}{llllll} \kappa_1 = 5, & \kappa_2 = 0.1, & \kappa_3 = 1, & \kappa_4 = 2, & \kappa_5 = 0.1, & \kappa_6 = 2, \\ \kappa_7 = 0.01, & \kappa_8 = 0.8, & \kappa_9 = 0.1, & \kappa_{10} = 0.01, & \kappa_{11} = 0.1. & \end{array} \tag{18}$$

1.8.1 The concentration $[S_p]$ as a function of $[S]$

First, we discuss how the concentration of S_p, x_6, changes according to the concentration of S, x_5, at steady state using (8).

Consider the expression (8) as a function of x_5:

$$x_6 = \varphi_6(x_5).$$

We are interested in the steady states for a fixed value of $S_{\text{tot}} = x_3 + x_4 + x_5 + x_6$. Therefore, we also consider the rational function of x_6 obtained by substitution of (6) ($x_3 = \varphi_3(x_5)$) and (7) ($x_4 = \varphi_4(x_5)$) into the conservation law for S_{tot}: $x_6 = S_{\text{tot}} - \varphi_3(x_5) - \varphi_4(x_5) - x_5$. This expresses x_6 by another rational function in x_5 whose numerator has degree three and denominator has degree two. We plot the two functions using the parameters in (18), $K_{\text{tot}} = 5$ and $S_{\text{tot}} = 591$.

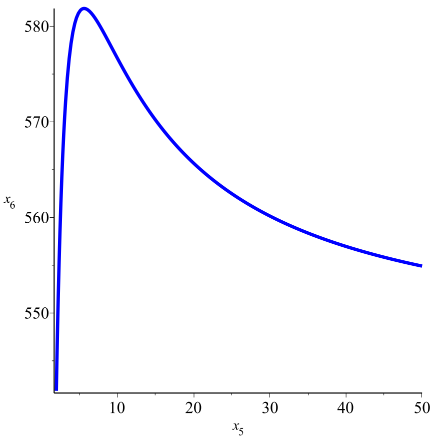

Figure 3a.

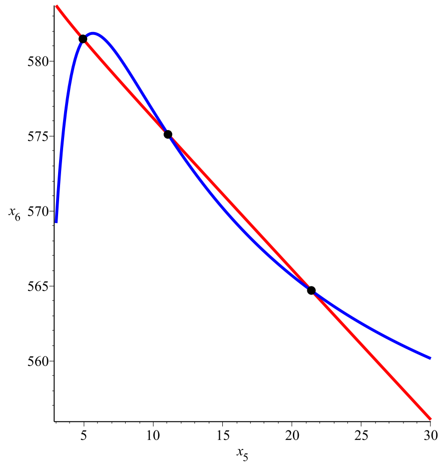

Figure 3b.

Figure 3a shows the graph of the function $\varphi_6(x_5)$. Figure 3b shows the graph of the function $\varphi_6(x_5)$ (in blue) together with the function $S_{\text{tot}} - \varphi_3(x_5) - \varphi_4(x_5) - x_5$ (in red). The intersection points of the two graphs in Figure 3b (marked with dots) are the pairs (x_5, x_6) for the three steady states corresponding to the chosen values of the total amounts K_{tot} and S_{tot}.

We observe in Figure 3a that $\varphi_6(x_5)$ increases for small values of x_5, until it reaches a maximum and then decreases towards a limit value for large x_5. In the next proposition we establish that this shape of $\varphi_6(x_5)$ is actually necessary for multistationarity.

Recall that by Proposition 4, for a given choice of reaction rate constants κ, there exist total amounts K_{tot} and S_{tot} such that the network admits multiple steady states if and only if $\alpha(\kappa) > 0$. We first list some properties of the function $\varphi_6(x_5)$.

Proposition 8. *Let the reaction rate constants κ and the total amount K_{tot} be given.*

- $\varphi_6(x_5) = 0$ *only if* $x_5 = 0$.
- *When x_5 tends to infinity, x_6 tends to the following limit value:*

$$\lim_{x_5 \to +\infty} \varphi_6(x_5) = \frac{K_{\text{tot}}\left(\kappa_3\kappa_{11} + \kappa_6\kappa_{10}\right)}{\kappa_7\left(\kappa_{10} + \kappa_{11}\right)}.$$

- *If $\alpha(\kappa) > 0$ (i.e. the network is multistationary), then there exists $\widehat{x}_5$ such that $\varphi_6(x_5)$ is increasing for $x_5 < \widehat{x}_5$ and is decreasing for $x_5 > \widehat{x}_5$.*
- *If $\alpha(\kappa) \leq 0$ (i.e. the network is not multistationary), then $\varphi_6(x_5)$ is either an increasing function for all x_5 or there exists $\widehat{x}_5$ such that $\varphi_6(x_5)$ is increasing for $x_5 < \widehat{x}_5$ and is decreasing for $x_5 > \widehat{x}_5$.*

Proof. It is straightforward to see that $\varphi_6(x_5) > 0$ unless $x_5 = 0$ and to compute the limit value using (8). The derivative of $\varphi_6(x_5)$ with respect to x_5 has the form

$$\varphi_6'(x_5) = \frac{K_{\text{tot}}\big(\lambda_1(\kappa)x_5^2 + \lambda_2(\kappa)x_5 + \lambda_3(\kappa)\big)}{\kappa_7^2 q(x_5)^2},$$

where $\lambda_2(\kappa), \lambda_3(\kappa)$ are positive polynomials in the reaction rate constants, and

$$\lambda_1(\kappa) = -\alpha(\kappa) - \overline{\lambda}_1(\kappa),$$

with $\alpha(\kappa)$ as in (12) and $\overline{\lambda}_1(\kappa)$ a positive polynomial in the reaction rate constants.

If $\lambda_1(\kappa)$ is positive, then $\varphi_6'(x_5) > 0$ for all $x_5 > 0$ and hence $\varphi_6(x_5)$ is an increasing function.

If $\lambda_1(\kappa)$ is negative, then the numerator of $\varphi_6'(x_5)$ is a second degree polynomial with negative leading coefficient $K_{\rm tot}\lambda_1(\kappa)$, and the rest of the coefficients are positive. This implies that the polynomial has a unique positive root, that is, there exists a unique $\widehat{x}_5 > 0$ for which the polynomial vanishes (and hence $\varphi_6'(\widehat{x}_5) = 0$). The numerator is negative for $x_5 > \widehat{x}_5$ and positive for $x_5 < \widehat{x}_5$. As a consequence, the derivative of $\varphi_6(x_5)$ is positive for $x_5 < \widehat{x}_5$ and negative for $x_5 > \widehat{x}_5$. It follows that $\varphi_6(x_5)$ is increasing for x_5 smaller than $\widehat{x}_5$ and is decreasing for x_5 larger than $\widehat{x}_5$.

When $\alpha(\kappa) > 0$, $\lambda_1(\kappa)$ is negative and hence $\varphi_6(x_5)$ has the shape stated in the proposition. If $\alpha(\kappa) \leq 0$, then $\lambda_1(\kappa)$ can either be negative or positive, giving the two options stated in the proposition. □

The proposition states that $x_6 = \varphi_6(x_5)$ settles at a non-zero value when x_5, and hence also $S_{\rm tot}$, are large (second statement in the proposition). When multistationarity occurs, Figure 3a illustrates that there are values of x_6 that correspond to two different values of x_5 (third statement in the proposition). It follows that we cannot express x_5 as a function of x_6, and hence we cannot express the other concentrations at steady state in terms of x_6 alone.

1.8.2 The concentrations $[K_r]$ and $[K_t]$ as functions of $[S]$

We consider the rational functions $x_1 = \varphi_1(x_5)$ in (4) and $x_2 = \varphi_2(x_5)$ in (5). The plot of these functions using the parameters in (18) and $K_{\rm tot} = 5$ are shown in Figure 4(a,b).

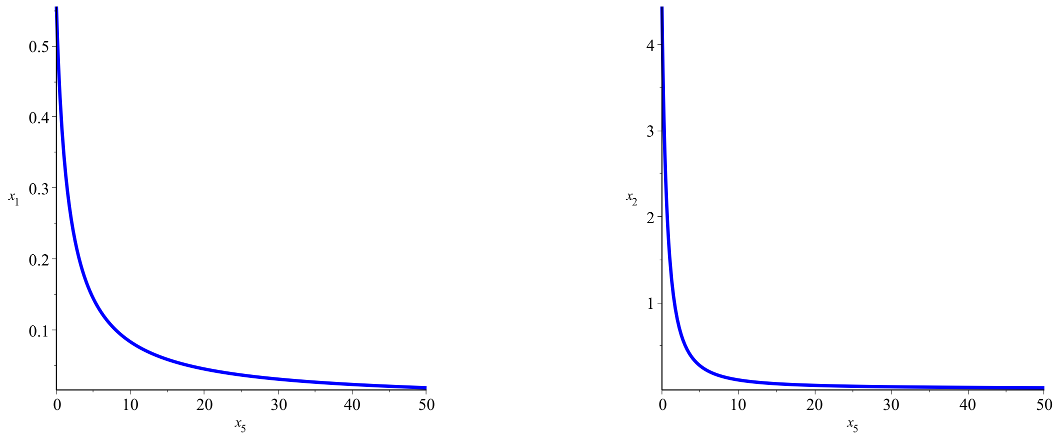

Figure 4a. Figure 4b.

Proposition 9. *Let the reaction rate constants κ and the total amount $K_{\rm tot}$ be given.*

- $\varphi_1(x_5) > 0$ *and* $\varphi_2(x_5) > 0$ *for all* $x_5 \geq 0$.
- $\varphi_1(x_5)$ *and* $\varphi_2(x_5)$ *tend to zero as* x_5 *tends to infinity.*
- *It holds for* $\varphi_1(x_5)$ *as well as* $\varphi_2(x_5)$ *that the function is either decreasing for all* x_5, *or there exists a value* $\widehat{x}_5$ *such that* $\varphi_3(x_5)$ *is increasing for* $x_5 < \widehat{x}_5$ *and is decreasing for* $x_5 > \widehat{x}_5$.

Proof. From (4) and (5), it follows that $\varphi_1(x_5) > 0$ and $\varphi_2(x_5) > 0$ for all $x_5 \geq 0$. Further, it follows that the numerator of both $\varphi_1(x_5)$ and $\varphi_2(x_5)$ has degree one and the denominator degree 2. Hence, $\varphi_1(x_5)$ and $\varphi_2(x_5)$ tend to zero as x_5 tends to infinity. The derivatives of $\varphi_1(x_5)$ and $\varphi_2(x_5)$ are of the form

$$\varphi_1'(x_5) = \frac{K_{\text{tot}}\big(a_1(\kappa)x_5^2 + a_2(\kappa)x_5 + a_3(\kappa)\big)}{q(x_5)^2}, \qquad \varphi_2'(x_5) = \frac{K_{\text{tot}}\big(b_1(\kappa)x_5^2 + b_2(\kappa)x_5 + b_3(\kappa)\big)}{q(x_5)^2},$$

where $a_1(\kappa), a_2(\kappa), b_1(\kappa), b_2(\kappa)$ are negative polynomials in the reaction rate constants, and

$$\begin{aligned} a_3(\kappa) &= -\overline{a}_3(\kappa)\kappa_4\kappa_8(\kappa_3+\kappa_2)(\kappa_9-\kappa_{11}) + \kappa_9((\kappa_1\kappa_9+\kappa_4\kappa_8)(\kappa_{10}+\kappa_{11}) + \kappa_1(\kappa_5+\kappa_6)(\kappa_{10}+\kappa_9)),\\ b_3(\kappa) &= -\overline{b}_3(\kappa)(\kappa_1\kappa_9(\kappa_8-\kappa_{10})(\kappa_5+\kappa_6) + \kappa_8(\kappa_4\left(\kappa_8+\kappa_{11}\right)\left(\kappa_2+\kappa_3\right)\\ &\qquad + \left(\kappa_{10}+\kappa_{11}\right)\left(\kappa_1\kappa_9+\kappa_4\kappa_8\right))), \end{aligned}$$

with $\overline{a}_3(\kappa)$ and $\overline{b}_3(\kappa)$ being positive polynomials in the reaction rate constants.

For small values of x_5, the sign of the derivative of $\varphi_1(x_5)$ and $\varphi_2(x_5)$ agrees with the sign of $a_3(\kappa)$ and $b_3(\kappa)$, respectively. If $a_3(\kappa)$ is negative, then $\varphi_1(x_5)$ decreases for all x_5. If $a_3(\kappa)$ is positive, then $\varphi_1'(x_5)$ vanishes at exactly one value $\widehat{x}_5$. In this case $\varphi_1(x_5)$ increases for $x_5 < \widehat{x}_5$ and decreases for $x_5 > \widehat{x}_5$. We argue analogously for the monotone behaviour of $\varphi_2(x_5)$ depending on the sign of $b_3(\kappa)$. □

1.8.3 The concentrations $[K_rS]$ and $[K_tS]$ as functions of $[S]$

We consider the rational functions $x_3 = \varphi_3(x_5)$ in (6) and $x_4 = \varphi_4(x_5)$ in (7). The plot of these functions using the parameters in (18) and $K_{\text{tot}} = 5$ are shown in Figure 5(a,b).

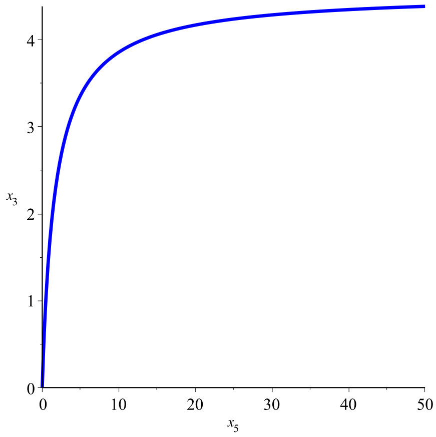

Figure 5a.

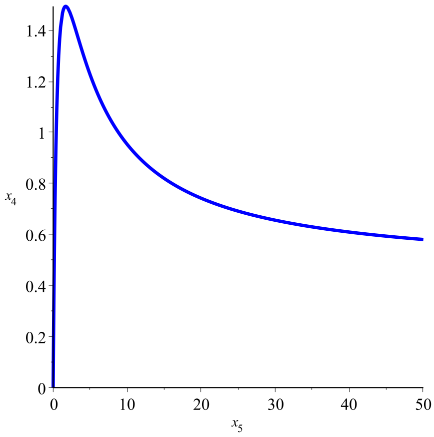

Figure 5b.

Proposition 10. *Let the reaction rate constants κ and the total amount K_{tot} be given.*

- $\varphi_3(x_5) = 0$ *only if* $x_5 = 0$, *and* $\varphi_4(x_5) = 0$ *only if* $x_5 = 0$.
- *We have*

$$\lim_{x_5\to+\infty} \varphi_3(x_5) = \frac{K_{\text{tot}}\kappa_{11}}{\kappa_{10}+\kappa_{11}}, \qquad \lim_{x_5\to+\infty} \varphi_4(x_5) = \frac{K_{\text{tot}}\kappa_{10}}{\kappa_{10}+\kappa_{11}}. \tag{19}$$

- *For both $\varphi_3(x_5)$ and $\varphi_4(x_5)$ it holds that: the function either increases for all x_5, or there exists a value $\widehat{x}_5$ such that $\varphi_3(x_5)$ increases for $x_5 < \widehat{x}_5$ and decreases for $x_5 > \widehat{x}_5$.*

Proof. From (6) and (7), it follows that $\varphi_3(x_5) = 0$ and $\varphi_4(x_5) = 0$ only if $x_5 = 0$. Further, it follows that the numerator and the denominator of both $\varphi_3(x_5)$ and $\varphi_4(x_5)$ have degree two. By inspecting the leading coefficient of the numerator and the denominator of these functions, we find the limit values given in the statement.

The derivatives of $\varphi_3(x_5)$ and $\varphi_4(x_5)$ are

$$\varphi_3'(x_5) = \frac{K_{\text{tot}}\big(c_1(\kappa)x_5^2 + c_2(\kappa)x_5 + c_3(\kappa)\big)}{q(x_5)^2}, \qquad \varphi_4'(x_5) = \frac{K_{\text{tot}}\big(d_1(\kappa)x_5^2 + d_2(\kappa)x_5 + d_3(\kappa)\big)}{q(x_5)^2},$$

where $c_2(\kappa), c_3(\kappa), d_2(\kappa), d_3(\kappa)$ are positive polynomials in the reaction rate constants, and

$$\begin{aligned} c_1(\kappa) &= \overline{c}_1(\kappa)(\kappa_1\kappa_{10}(\kappa_{11} - \kappa_9)(\kappa_5 + \kappa_6) + \kappa_4\kappa_{11}(\kappa_8 + \kappa_{11})(\kappa_2 + \kappa_3)), \\ d_1(\kappa) &= \overline{d}_1(\kappa)(\kappa_4\kappa_{11}(\kappa_{10} - \kappa_8)(\kappa_2 + \kappa_3) + \kappa_1\kappa_{10}(\kappa_{10} + \kappa_9)(\kappa_5 + \kappa_6)), \end{aligned}$$

with $\overline{c}_1(\kappa)$ and $\overline{d}_1(\kappa)$ positive polynomials in the reaction rate constants. The only coefficients in the numerators of $\varphi_3(x_5)$ and $\varphi_4(x_5)$ that have undetermined sign are thus $c_1(\kappa)$ and $d_1(\kappa)$ and the derivatives can change sign at most once. It follows that for small values of x_5, both derivatives take positive values and the functions are increasing. For large x_5, the derivatives are positive or negative, depending on the signs of $c_1(\kappa)$ and $d_1(\kappa)$ (and thus on the reaction rate constants). Therefore, each of the functions $\varphi_3(x_5)$ and $\varphi_4(x_5)$ can either be increasing towards the limit (19) (as in Figure 5a) or be increasing towards a maximum value and then be decreasing towards the limit in (19) (as in Figure 5b). □

These results show that for a steady state with a large concentration of S, the concentrations of K_rS and K_tS are close to a limit value, and the concentrations of K_r and K_t are close to zero. This confirms mathematically that saturation occurs: large amounts of substrate imply that the kinase is essentially only in bound form.

1.9 Bifurcation plots with K_{tot} and S_{tot}

In this subsection we investigate how the number of positive steady states depends on the total amounts of kinase and substrate.

At steady state, $p(x_5) = 0$. The polynomial $p(x_5)$ is linear in S_{tot} and in K_{tot}. Hence, we can use the equation $p(x_5) = 0$ to isolate S_{tot} (resp. K_{tot}) and get an expression of S_{tot} (resp. K_{tot}) as a function of x_5, the reaction rate constants and K_{tot} (resp. S_{tot}). In this way we obtain two functions

$$\begin{aligned} \psi_S(x_5) = \Big(&\kappa_1\kappa_4\kappa_7(\kappa_{10} + \kappa_{11})x_5^3 + (K_{\text{tot}}\kappa_1\kappa_4((\kappa_3 + \kappa_7)\kappa_{11} + (\kappa_6 + \kappa_7)\kappa_{10}) \\ &+ \kappa_7(\kappa_1(\kappa_5 + \kappa_6)(\kappa_9 + \kappa_{10}) + (\kappa_1\kappa_9 + \kappa_4\kappa_8)(\kappa_{10} + \kappa_{11}) + \kappa_4(\kappa_2 + \kappa_3)(\kappa_8 + \kappa_{11})))x_5^2 \\ &+ (K_{\text{tot}}((\kappa_2 + \kappa_3)\kappa_4(\kappa_6 + \kappa_7)\kappa_8 + \kappa_1(\kappa_3 + \kappa_7)(\kappa_5 + \kappa_6)\kappa_9 \\ &+ \kappa_7(\kappa_1\kappa_9 + \kappa_4\kappa_8)(\kappa_{10} + \kappa_{11}) + (\kappa_3\kappa_{11} + \kappa_6\kappa_{10})(\kappa_1\kappa_9 + \kappa_4\kappa_8)) \\ &+ \kappa_7(\kappa_8 + \kappa_9)((\kappa_2 + \kappa_3 + \kappa_{10})(\kappa_5 + \kappa_6) + (\kappa_2 + \kappa_3)\kappa_{11}))x_5\Big)\Big/ \\ \Big(&\kappa_1\kappa_4\kappa_7(\kappa_{10} + \kappa_{11})x_5^2 + \kappa_7(\kappa_1(\kappa_5 + \kappa_6)(\kappa_9 + \kappa_{10}) + (\kappa_1\kappa_9 + \kappa_4\kappa_8)(\kappa_{10} + \kappa_{11}) \\ &+ (\kappa_2 + \kappa_3)\kappa_4(\kappa_8 + \kappa_{11}))x_5 + \kappa_7(\kappa_8 + \kappa_9)((\kappa_2 + \kappa_3)(\kappa_5 + \kappa_6 + \kappa_{11}) + (\kappa_5 + \kappa_6)\kappa_{10})\Big) \end{aligned} \tag{20}$$

and

$$\begin{aligned}
\psi_K(x_5) = &\kappa_7\Big(-\kappa_1\kappa_4(\kappa_{10}+\kappa_{11})x_5^3 + (S_{\text{tot}}\kappa_1\kappa_4(\kappa_{10}+\kappa_{11}) - \kappa_1(\kappa_5+\kappa_6)(\kappa_9+\kappa_{10}) \\
&- (\kappa_1\kappa_9+\kappa_4\kappa_8)(\kappa_{10}+\kappa_{11}) - (\kappa_2+\kappa_3)\kappa_4(\kappa_8+\kappa_{11}))x_5^2 \\
&+ (S_{\text{tot}}(\kappa_1(\kappa_5+\kappa_6)(\kappa_9+\kappa_{10}) + (\kappa_1\kappa_9+\kappa_4\kappa_8)(\kappa_{10}+\kappa_{11}) + (\kappa_2+\kappa_3)\kappa_4(\kappa_8+\kappa_{11})) \\
&- (\kappa_8+\kappa_9)((\kappa_2+\kappa_3+\kappa_{10})(\kappa_5+\kappa_6) + (\kappa_2+\kappa_3)\kappa_{11}))x_5 \\
&+ S_{\text{tot}}(\kappa_8+\kappa_9)((\kappa_2+\kappa_3+\kappa_{10})(\kappa_5+\kappa_6) + (\kappa_2+\kappa_3)\kappa_{11})\Big) \Big/ \\
&\Big(\kappa_1\kappa_4((\kappa_3+\kappa_7)\kappa_{11} + (\kappa_6+\kappa_7)\kappa_{10})x_5^2 + (\kappa_4\kappa_8((\kappa_2+\kappa_3+\kappa_{10})(\kappa_6+\kappa_7) + (\kappa_3+\kappa_7)\kappa_{11}) \\
&+ \kappa_1\kappa_9((\kappa_3+\kappa_7)(\kappa_5+\kappa_6+\kappa_{11}) + (\kappa_6+\kappa_7)\kappa_{10}))x_5\Big),
\end{aligned} \tag{21}$$

such that

$$S_{\text{tot}} = \psi_S(x_5) \quad \text{and} \quad K_{\text{tot}} = \psi_K(x_5)$$

hold at steady state. In what follows we study the shape of these two functions when multistationarity occurs.

1.9.1 Changing S_{tot}

We fix first the total amount of kinase K_{tot} and analyse $\psi_S(x_5)$. We start with an example. We let $K_{\text{tot}} = 5$ and choose the reaction rate constants as in (18). We plot the value of S_{tot} against x_5 at steady state. Figure 6b is the graph of $\psi_S(x_5)$, while Figure 6a is the bifurcation plot obtained by interchanging the axes of the plot in Figure 6b.

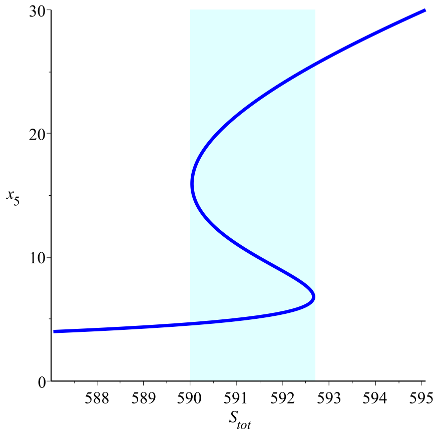

Figure 6a.

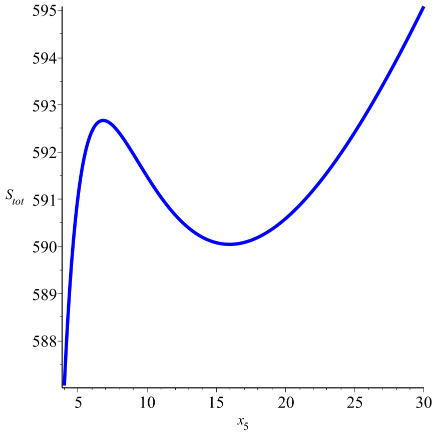

Figure 6b.

From Figure 6a, we conclude that multistationarity occurs for values of S_{tot} in a certain interval (highlighted in blue in the figure): the values of S_{tot} that correspond to three values of x_5 (which in turn give rise to three positive steady states). For low values of S_{tot} the concentration of x_5 is low and for higher values of S_{tot} the concentration of x_5 is high.

To understand Figure 6a, we study the function $\psi_S(x_5)$ in Figure 6b, since the bifurcation plot is simply obtained by interchanging the axes. We do this because we do not have an analytical expression of the type $x_5 = \Phi(S_{\text{tot}})$.

Proposition 11. *Fix the reaction rate constants κ and the total amount K_{tot}.*

- $\psi_S(x_5) = 0$ *when* $x_5 = 0$ *and* $\psi_S(x_5)$ *tends to infinity as* x_5 *goes to infinity.*
- *If there exists* S_{tot} *such that the network has multiple positive steady states, then* $\psi_S(x_5)$ *has exactly one local maximum, one local minimum and the monotonicity pattern of* $\psi_S(x_5)$ *is increasing-decreasing-increasing (as shown in Figure 6b).*
- *If the network does not admit multiple positive steady states for any* S_{tot}*, then* $\psi_S(x_5)$ *is an increasing function.*
- *If* $\alpha(\kappa) > 0$*, then the network admits multiple positive steady states for some* S_{tot}*, provided* K_{tot} *is large enough.*

Proof. The function $\psi_S(x_5)$ is a rational function whose numerator has degree three and the denominator has degree two. The coefficients of the numerator and denominator of $\psi_S(x_5)$ are positive polynomials in the reaction rate constants and K_{tot}. Hence, $\psi_S(x_5)$ tends to infinity as x_5 goes to infinity.

The derivative of $\psi_S(x_5)$ with respect to x_5 is the rational function

$$\psi_S'(x_5) = \frac{\mu_1(\kappa)x_5^4 + \mu_2(\kappa)x_5^3 + \mu_3(\kappa, K_{\text{tot}})x_5^2 + \mu_4(\kappa, K_{\text{tot}})x_5 + \mu_5(\kappa, K_{\text{tot}})}{q_2(\kappa, x_5)},$$

where $\mu_1(\kappa)$ and $\mu_2(\kappa)$ are positive polynomials in the reaction rate constants, $\mu_4(\kappa, K_{\text{tot}})$ and $\mu_5(\kappa, K_{\text{tot}})$ are positive polynomials in the reaction rate constants and K_{tot} (depending linearly on K_{tot}), $q_2(\kappa, x_5)$ is a degree 4 polynomial in x_5 whose coefficients are positive polynomials in the reaction rate constants and

$$\mu_3(\kappa, K_{\text{tot}}) = \overline{\mu}_3(\kappa) - K_{\text{tot}}\alpha(\kappa),$$

with $\overline{\mu}_3(\kappa)$ a positive polynomial in the reaction rate constants and $\alpha(\kappa)$ as in (12). Thus the only coefficient of the numerator of $\psi_S'(x_5)$ that can be negative is $\mu_3(\kappa, K_{\text{tot}})$.

Since the independent and leading coefficients of the numerator of $\psi_S'(x_5)$ are positive, $\psi_S'(x_5)$ is positive for small and large enough values of x_5 and the function $\psi_S(x_5)$ is increasing.

Multistationarity occurs if and only if there are values of x_5 for which the corresponding values of $S_{\text{tot}} = \psi_S(x_5)$ agree. This occurs if and only if $\psi_S(x_5)$ decreases in some interval (it cannot be an increasing function) and hence necessarily $\mu_3(\kappa, K_{\text{tot}})$ is negative. In this case the sequence of coefficients of the polynomial in the numerator of $\psi_S'(x_5)$ has two changes of sign and the Descartes' rule of signs tells us that $\psi_S'(x_5) = 0$ has at most two solutions. It follows that the function $\psi_S(x_5)$ cannot change the increasing/decreasing behaviour more than twice. Combined with the discussion above, multistationarity implies that the monotonicity pattern of $\psi_S(x_5)$ is increasing-decreasing-increasing. We deduce that there is exactly one local maximum and one local minimum when the system displays multistationarity and that the network has three steady states (and not more). We conclude that the graph of $\psi_S(x_5)$ must be as in Figure 6b when multistationarity occurs, that is, it has an S-shape.

Since $\mu_1(\kappa), \mu_2(\kappa)$ do not depend on K_{tot} and $\mu_3(\kappa, K_{\text{tot}}), \mu_4(\kappa, K_{\text{tot}}), \mu_5(\kappa, K_{\text{tot}})$ depend linearly on K_{tot}, we deduce that if $\alpha(\kappa) > 0$ there are always values of K_{tot} such that $\psi_S'(x_5)$ is negative for some values of x_5 (for K_{tot} large enough such that $-K_{\text{tot}}\alpha(\kappa)x_5^2$ dominates). Therefore, we see again that $\alpha(\kappa) > 0$ is a necessary and sufficient condition for multistationarity. □

We might also plot S_{tot} against x_6 by plotting $(S_{\text{tot}}, x_6) = (\psi_S(x_5), \varphi_6(x_5))$, for varying values of x_5:

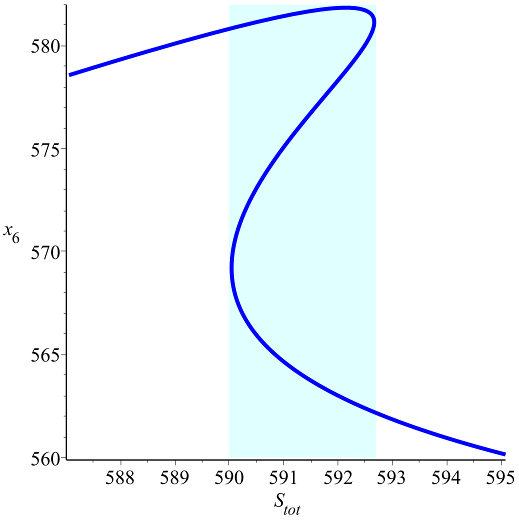

Figure 7a.

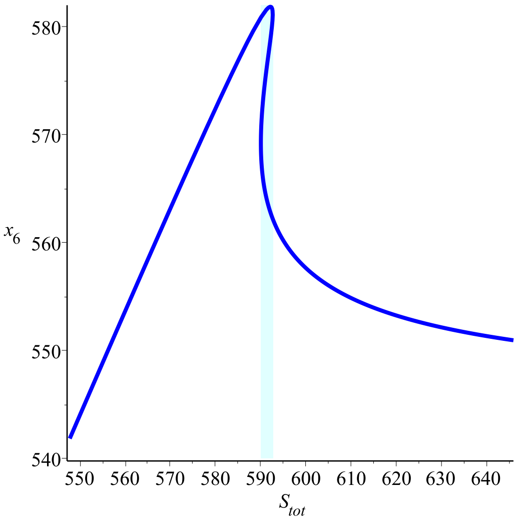

Figure 7b.

In Figure 7a, the values of S_{tot} for which multistationarity occurs can be identified (highlighted in blue in the figure). In Figure 7b, we used a larger range of x_5 values in the plot. We find that x_6 increases with S_{tot} towards a maximum value after which x_6 decreases. After the maximum value there is a transition phase where multistationarity occurs, and finally for larger S_{tot} there is one steady state. In this case it is not straightforward to pursue a general analysis of the relationship between S_{tot} and x_6, as we did for S_{tot} and x_5, because we cannot express x_5, and hence S_{tot}, as a function of x_6.

1.9.2 Changing K_{tot}

We consider the value of S_{tot} fixed and analyse $K_{\text{tot}} = \psi_K(x_5)$. Figure 8 shows the bifurcation plot obtained by plotting the function $\psi_K(x_5)$ with $S_{\text{tot}} = 591$ and the reaction rate constants as in (18), with the axes interchanged. The region with three positive steady states is highlighted in blue.

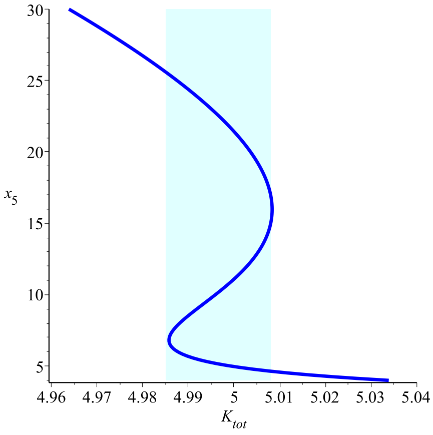

Figure 8.

To investigate the behaviour of $\psi_K(x_5)$, we plot the function $\psi_K(x_5)$ for two different domains of x_5, see Figure 9(a,b).

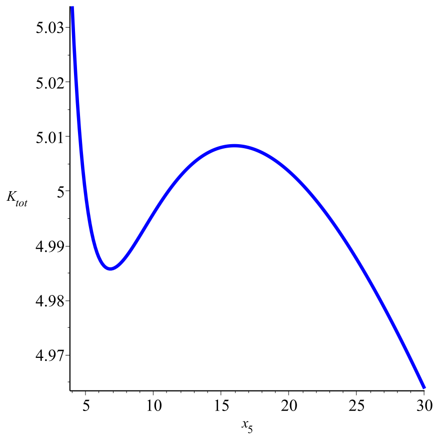

Figure 9a.

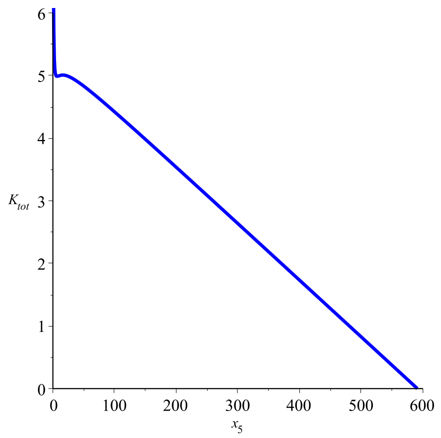

Figure 9b.

Proposition 12. *Fix the reaction rate constants κ and the total amount S_{tot}.*

- *$\psi_K(x_5)$ is well defined and continuous for $x_5 > 0$, tends to plus infinity as x_5 tends to zero, is positive only when $0 < x_5 < S_{\text{tot}}$ and it holds that $\psi_K(S_{\text{tot}}) = 0$.*
- *If there exists K_{tot} such that the network has multiple positive steady states, then $\psi_K(x_5)$ has exactly one local maximum, one local minimum and the monotonicity pattern of $\psi_K(x_5)$ is decreasing-increasing-decreasing (as shown in Figure 9a).*
- *If the network does not admit multiple positive steady states for any K_{tot}, then $\psi_K(x_5)$ is a decreasing function.*
- *If $\alpha(\kappa) > 0$, then the network admits multiple positive steady states for some K_{tot}, provided S_{tot} is large enough.*

Proof. The function $\psi_K(x_5)$ is a rational function whose numerator has degree three and the denominator has degree two (see (21)). The denominator of $\psi_K(x_5)$ vanishes when $x_5 = 0$ and hence $\psi_K(x_5)$ is only defined and continuous for $x_5 > 0$. Since the independent term of the numerator is positive, we deduce that $\psi_K(x_5)$ (and thus K_{tot}) tends to plus infinity as x_5 tends to zero. We next analyse when $\psi_K(x_5)$ is positive. The denominator of $\psi_K(S_{\text{tot}})$ is positive for all strictly positive values of x_5. It is easy to see that $\psi_K(S_{\text{tot}}) = 0$. The sequence of coefficients of the polynomial in the numerator of $\psi_K(x_5)$ has exactly one change of sign. Thus by Descartes' rule of signs, $x_5 = S_{\text{tot}}$ is the only positive root of the numerator. Since the independent term of the numerator is positive, the function $\psi_K(x_5)$ is positive for $x_5 < S_{\text{tot}}$ and negative for $x_5 > S_{\text{tot}}$.

The derivative of $\psi_K(x_5)$ with respect to x_5 is a rational function

$$\psi_K'(x_5) = \frac{\gamma_1(\kappa)x_5^4 + \gamma_2(\kappa)x_5^3 + \gamma_3(\kappa, S_{\text{tot}})x_5^2 + \gamma_4(\kappa, S_{\text{tot}})x_5 + \gamma_5(\kappa, S_{\text{tot}})}{q_3(\kappa, x_5)},$$

where $\gamma_1(\kappa), \gamma_2(\kappa)$ are negative polynomials in the reaction rate constants, $\gamma_4(\kappa, S_{\text{tot}}), \gamma_5(\kappa, S_{\text{tot}})$ are negative polynomials in the reaction rate constants and S_{tot} (they are linear in S_{tot}),

$q_3(\kappa, x_5)$ is a degree 4 polynomial in x_5 whose coefficients are positive polynomials in the reaction rate constants, and

$$\gamma_3(\kappa, S_{\text{tot}}) = S_{\text{tot}}\kappa_7\alpha(\kappa) + \overline{\gamma}_3(\kappa),$$

where $\overline{\gamma}_3(\kappa)$ is a polynomial in the reaction rate constants with positive and negative terms, and $\alpha(\kappa)$ is as in (12).

The only coefficient of the numerator of $\psi_K'(x_5)$ that can have both signs is $\gamma_3(\kappa, S_{\text{tot}})$. Since the leading and independent terms $\gamma_1(\kappa)$ and $\gamma_5(\kappa, S_{\text{tot}})$ are negative, we deduce that $\psi_K'(x_5)$ is negative for small and large enough values of x_5. Thus $\psi_K(x_5)$ decreases in this case.

If multistationarity occurs for some values of K_{tot}, $\psi_K(x_5)$ must increase in some interval, where necessarily $\gamma_3(\kappa, S_{\text{tot}}) > 0$. We argue as above for $\psi_S(x_5)$ to conclude that the function has exactly one local maximum and one local minimum. In this case the graph of $\psi_K(x_5)$ has the same S-shape as in the example graph in Figure 9a. If multistationarity does not occur, then $\psi_K(x_5)$ must be decreasing for all $0 < x_5 \leq S_{\text{tot}}$.

Since $\gamma_1(\kappa), \gamma_2(\kappa)$ do not depend on S_{tot} and $\gamma_3(\kappa, S_{\text{tot}}), \gamma_4(\kappa, S_{\text{tot}}), \gamma_5(\kappa, S_{\text{tot}})$ depend linearly on S_{tot}, we deduce that if $\alpha(\kappa) > 0$, then there are always values of S_{tot} that make $\psi_K'(x_5)$ negative for certain values of x_5 (for S_{tot} large enough such that $S_{\text{tot}}\kappa_7\alpha(\kappa)x_5^2$ dominates). Therefore, we see once again that $\alpha(\kappa) > 0$ is a necessary and sufficient condition for multistationarity. □

The first statement of the proposition tells us that the function $\psi_K(x_5)$ needs to be considered for $0 < x_5 \leq S_{\text{tot}}$. K_{tot} tends to infinity as x_5 tends to zero and is zero when $x_5 = S_{\text{tot}}$. This implies that the concentration of S at steady state, x_5, equals S_{tot} when $K_{\text{tot}} = 0$ and tends to zero as K_{tot} tends to infinity.

We plot also the value of x_6 against K_{tot}. To do that, we use the expression of x_6 given in (8), where we substitute K_{tot} with $\psi_K(x_5)$. Then we plot the pair of functions (K_{tot}, x_6) for varying values of x_5:

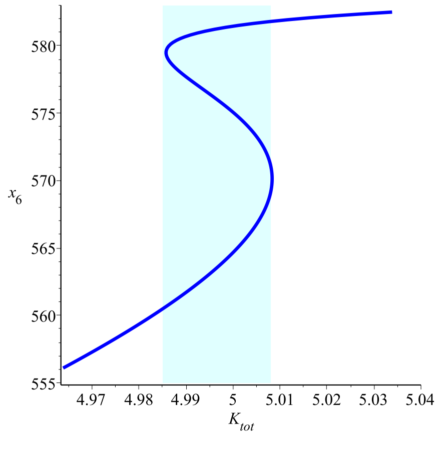

Figure 10a.

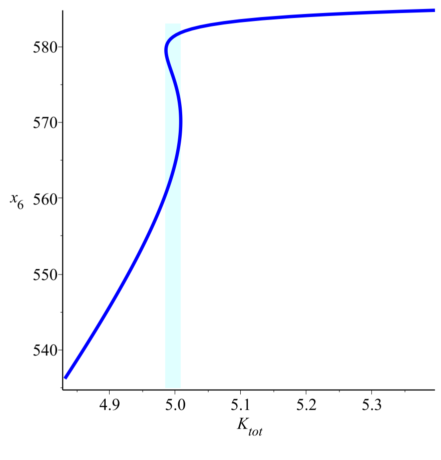

Figure 10b.

For low values of K_{tot}, the concentration of x_6 increases, until it reaches a transitional phase, after which it tends towards an upper bound, the total amount of substrate S_{tot}. This bound is found by using that $x_5 = 0$ when K_{tot} tends to infinity. Specifically, it is found by letting $x_5 = 0$ in the expression of x_6 given by (8) after replacing K_{tot} by $\psi_K(x_5)$.

1.10 Positive feedback loops

The following figure shows the DSR-graph (directed-species-reaction graph) for the network in Subsection 1.1. The DSR-graph is constructed as follows: The node set is

$$\{K_r, K_t, K_rS, K_tS, S, S_p, R_1, \ldots, R_{11}\}.$$

There is an edge from a species X to a reaction R with label $+$ if X is a reactant of R. There is an edge from a reaction R to a species X if the net production of X in R is different from zero. The label is $+$ if the net production is positive and $-$ otherwise.

We highlight in green and red the four positive feedback loops that contribute to multistationarity, as found following the method in [5].

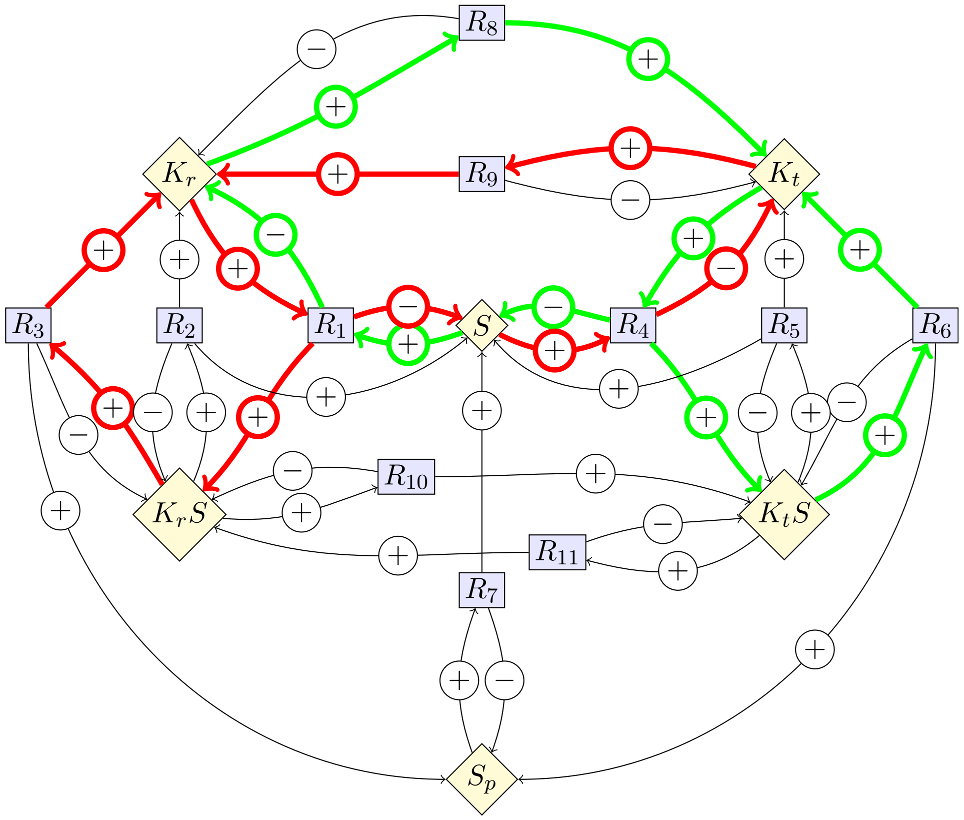

2 The core model for n allosteric kinase competing for the same substrate

In this section we consider a generalisation of the model studied in the previous section. Namely, we consider the network that consists of n allosteric kinases that act on the same substrate. The network studied in the previous section corresponds to the case $n = 1$.

We show that the network with n allosteric kinases admits $2n + 1$ steady states for some choice of reaction rate constants and total amounts. In order to prove this, we use the reduction techniques explained in subsection 1.3. Explicitly, we make some reversible reactions irreversible and make use of Theorem 2.

For $n = 1$ the reduction corresponds to setting $\kappa_2 = \kappa_5 = 0$ and $\kappa_9 = \kappa_{10} = 0$ in the model in subsection 1.1. Biologically, we can interpret the first choice as assuming strong complex formation. The second choice is compatible with condition (11). Since we aim to study the effect of multiple kinases on multistationarity, we consider conditions that are compatible with multistationarity in the single kinase case. This reduced model is still multistationary as we will show below.

2.1 Model description

We study a reduction of the system consisting of n allosteric kinases competing for the same substrate.

Let K^i, for $i = 1, \ldots, n$, denote the n allosteric kinases. We use subindices r, t to denote the relaxed or tensed state (respectively) of each of them. The set of reactions given in the previous subsection are reproduced for the n allosteric kinases, after making some reversible reactions irreversible and renaming the reaction rate constants accordingly. That is, for $i = 1, \ldots, n$, the reactions under consideration are as follows:

$$K_r^i + S \xrightarrow{\kappa_{i,1}} K_r^i S \xrightarrow{\kappa_{i,2}} K_r^i + S_p \qquad\qquad K_r^i \xrightarrow{\kappa_{i,5}} K_t^i$$
$$K_t^i + S \xrightarrow{\kappa_{i,3}} K_t^i S \xrightarrow{\kappa_{i,4}} K_t^i + S_p \qquad\qquad K_t^i S \xrightarrow{\kappa_{i,6}} K_r^i S.$$

In addition, there is a dephosphorylation reaction

$$S_p \xrightarrow{\kappa_7} S.$$

In the full model, the reactions with reaction rate constants $\kappa_{i,1}$, $\kappa_{i,3}$, $\kappa_{i,5}$ and $\kappa_{i,6}$ are reversible.

We denote the concentration of the species as follows:

$$x_{i,1} := [K_r^i] \quad x_{i,2} := [K_t^i] \quad x_{i,3} := [K_r^i S] \quad x_{i,4} := [K_t^i S] \quad x_5 := [S] \quad x_6 := [S_p],$$

for $i = 1, \ldots, n$. We proceed as in the previous section and model the dynamics of the concentrations over time under the law of mass action by the following system of ordinary

differential equations:

$$\begin{aligned}
\dot{x}_{i,1} &= -\kappa_{i,1}x_{i,1}x_5 + \kappa_{i,2}x_{i,3} - \kappa_{i,5}x_{i,1} \\
\dot{x}_{i,2} &= -\kappa_{i,3}x_{i,2}x_5 + \kappa_{i,4}x_{i,4} + \kappa_{i,5}x_{i,1} \\
\dot{x}_{i,3} &= \kappa_{i,1}x_{i,1}x_5 - \kappa_{i,2}x_{i,3} + \kappa_{i,6}x_{i,4} \\
\dot{x}_{i,4} &= \kappa_{i,3}x_{i,2}x_5 - \kappa_{i,4}x_{i,4} - \kappa_{i,6}x_{i,4} \\
\dot{x}_5 &= -\sum_{j=1}^{n}\left(\kappa_{j,1}x_{j,1}x_5 + \kappa_{j,3}x_{j,2}x_5\right) + \kappa_7 x_6 \\
\dot{x}_6 &= \sum_{j=1}^{n}\left(\kappa_{j,2}x_{j,3} + \kappa_{j,4}x_{j,4}\right) - \kappa_7 x_6
\end{aligned}$$

for $i = 1, \dots, n$. The system has $n+1$ conservation laws: one for the substrate and one for each kinase. Namely, for $i = 1, \dots, n$, we have

$$x_{i,1} + x_{i,2} + x_{i,3} + x_{i,4} = K^i_{\text{tot}} \tag{22}$$

for some $K^i_{\text{tot}} > 0$, and for $S_{\text{tot}} > 0$,

$$x_5 + x_6 + \sum_{i=1}^{n}(x_{i,3} + x_{i,4}) = S_{\text{tot}}. \tag{23}$$

2.2 Summary of results

The results for the reduced model with n allosteric kinases competing for the same substrate can be summarised in the following way. In subsection 2.3 we show that the steady states of the system can be given in terms of the concentration x_5 of the substrate S only. That is, the concentrations of the other species at steady state can be found from the value of x_5 at steady state.

In subsection 2.4, we show that there exist reaction rate constants and total amounts such that the system has *exactly* $2m+1$ positive steady states, for all $m = 0, \dots, n$. In particular this is true for $m = n$ in which case there are $2n+1$ positive steady states. We show that $2n+1$ is the maximal possible number of non-negative steady states (each concentration is positive or zero). In subsection 2.5 we consider the stability of the steady states and show that if there are $2n+1$ positive steady states, then at least n of them are unstable.

These results extend to the full model (with the reactions with reaction rate constants $\kappa_{i,1}$, $\kappa_{i,3}$, $\kappa_{i,5}$ and $\kappa_{i,6}$ being reversible), due to Theorem 2.

2.3 Positive steady states

The positive steady states of the system are the solutions to the equations $\dot{x}_{i,1} = \dot{x}_{i,2} = \dot{x}_{i,3} = \dot{x}_{i,4} = 0$, for $i = 1, \dots, n$, together with $\dot{x}_5 = \dot{x}_6 = 0$, constrained by the conservation laws (22) and (23). We reason as in the previous section and disregard the steady state equations $\dot{x}_5 = 0$ and $\dot{x}_{i,1} = 0$, for $i = 1, \dots, n$. Using the equations $\dot{x}_{i,2} = \dot{x}_{i,3} = \dot{x}_{i,4} = 0$ and (22), we obtain algebraic expressions for $x_{i,1}, x_{i,2}, x_{i,3}, x_{i,4}$ at steady state, depending on the value of

x_5 at steady state, analogous to the expressions (4)-(7):

$$x_{i,1} = \frac{K^i_{\text{tot}}\kappa_{i,2}\kappa_{i,3}\kappa_{i,6}x_5}{q_i(x)} \tag{24}$$

$$x_{i,2} = \frac{K^i_{\text{tot}}(\kappa_{i,4}+\kappa_{i,6})\kappa_{i,2}\kappa_{i,5}}{q_i(x)} \tag{25}$$

$$x_{i,3} = \frac{K^i_{\text{tot}}\kappa_{i,3}\kappa_{i,6}(\kappa_{i,1}x_5+\kappa_{i,5})x_5}{q_i(x)} \tag{26}$$

$$x_{i,4} = \frac{K^i_{\text{tot}}\kappa_{i,2}\kappa_{i,3}\kappa_{i,5}x_5}{q_i(x)} \tag{27}$$

where $q_i(x)$ is the following polynomial in x_5:

$$q_i(x) = \kappa_{i,1}\kappa_{i,3}\kappa_{i,6}x_5^2 + \kappa_{i,3}(\kappa_{i,2}\kappa_{i,5}+\kappa_{i,2}\kappa_{i,6}+\kappa_{i,5}\kappa_{i,6})x_5 + \kappa_{i,2}\kappa_{i,5}(\kappa_{i,4}+\kappa_{i,6}).$$

These expressions are positive provided x_5 is positive. From the equation $\dot{x}_6 = 0$ we obtain

$$x_6 = \frac{\sum_{j=1}^n(\kappa_{j,2}x_{j,3}+\kappa_{j,4}x_{j,4})}{\kappa_7} \tag{28}$$

which, using expressions (26) and (27), is positive provided $x_5 > 0$.

All concentrations are expressed as functions of x_5. We replace x_6 and subsequently $x_{i,3}, x_{i,4}$ in (23) by their expressions in (26)-(28) to obtain

$$(x_5 - S_{\text{tot}}) + \sum_{i=1}^n \frac{K^i_{\text{tot}}x_5\kappa_{i,3}((1+\kappa_{i,2}/\kappa_7)\,\kappa_{i,6}(\kappa_{i,1}x_5+\kappa_{i,5}) + (1+\kappa_{i,4}/\kappa_7)\,\kappa_{i,2}\kappa_{i,5})}{q_i(x)} = 0. \tag{29}$$

After multiplying this equation by $\prod_{i=1}^n q_i(x)$, the left-hand side becomes a polynomial $p(x_5)$ of degree $2n+1$ in x_5. As argued in the previous section, any positive root of the polynomial corresponds to a positive steady state. We note again that $p(x_5)$ has at least one positive root since one can check that $p(0) < 0$ and $p(+\infty) > 0$.

2.4 Existence of $2n+1$ positive steady states.

We have shown that the positive steady states of the reduced system with n allosteric kinases competing for the same substrate are determined by the positive solutions to a polynomial $p(x_5)$ of degree $2n+1$. By the fundamental theorem of algebra, a polynomial of degree $2n+1$ has $2n+1$ complex roots counted with multiplicity. Therefore, such a polynomial can *at most* have $2n+1$ distinct positive real roots.

We show in this section that there exist choices of reaction rate constants κ_i and total amounts $K^i_{\text{tot}}, S_{\text{tot}}$ such that the polynomial has exactly $2n+1$ distinct positive real roots. As a consequence, this proves that the reduced system with n allosteric kinases competing for the same substrate admits $2n+1$ positive steady states for some choice of reaction rate constants and total amounts and there cannot be more. As argued at the beginning of the section, Theorem 2 guarantees that this result holds for the general system, where the conformational changes of the kinases and the binding of the kinases and the substrate are considered reversible.

The proof of this statement consists of a series of simplifications and constructions analogous to those in [7].

First of all, observe that the steady states of the system are invariant by multiplication of all reaction rate constants by some scalar $\lambda > 0$. Therefore, we can assume that $\kappa_7 = 1$. For simplicity we write x for x_5 (not to be confused with x denoting the vector of concentrations). We let

$$\alpha_{i,1} = (\kappa_{i,2} + 1)\, K^i_{\text{tot}} \kappa_{i,1} \kappa_{i,3} \kappa_{i,6} \tag{30}$$
$$\alpha_{i,2} = K^i_{\text{tot}} \kappa_{i,3} \kappa_{i,5} ((\kappa_{i,2} + 1)\, \kappa_{i,6} + (\kappa_{i,4} + 1)\, \kappa_{i,2}) \tag{31}$$
$$\alpha_{i,3} = \kappa_{i,1} \kappa_{i,3} \kappa_{i,6} \tag{32}$$
$$\alpha_{i,4} = \kappa_{i,3} \left(\kappa_{i,2} \kappa_{i,5} + \kappa_{i,2} \kappa_{i,6} + \kappa_{i,5} \kappa_{i,6}\right) \tag{33}$$
$$\alpha_{i,5} = \kappa_{i,2} \kappa_{i,5} \left(\kappa_{i,4} + \kappa_{i,6}\right), \tag{34}$$

such that we write the terms in the sum in (29) as

$$\frac{K^i_{\text{tot}} x_5 \kappa_{i,3} ((1 + \kappa_{i,2})\, \kappa_{i,6} (\kappa_{i,1} x_5 + \kappa_{i,5}) + (1 + \kappa_{i,4})\, \kappa_{i,2} \kappa_{i,5})}{q_i(x)} = \frac{\alpha_{i,1} x^2 + \alpha_{i,2} x}{\alpha_{i,3} x^2 + \alpha_{i,4} x + \alpha_{i,5}}.$$

Lemma 1. *For any positive values $\alpha_{i,1}, \dots, \alpha_{i,5} > 0$, there exist $\kappa_{i,1}, \dots, \kappa_{i,6} > 0$ and $K^i_{\text{tot}} > 0$ such that* (30)-(34) *are fulfilled.*

Proof. To simplify the notation, we prove that for all $\alpha_1, \dots, \alpha_5 > 0$ there exist $\kappa_1, \dots, \kappa_6, K_{\text{tot}} > 0$ such that

$$\alpha_1 = (\kappa_2 + 1)\, K_{\text{tot}} \kappa_1 \kappa_3 \kappa_6 \tag{35}$$
$$\alpha_2 = K_{\text{tot}} \kappa_3 \kappa_5 ((\kappa_2 + 1)\, \kappa_6 + (\kappa_4 + 1)\, \kappa_2) \tag{36}$$
$$\alpha_3 = \kappa_1 \kappa_3 \kappa_6 \tag{37}$$
$$\alpha_4 = \kappa_3 \left(\kappa_2 \kappa_5 + \kappa_2 \kappa_6 + \kappa_5 \kappa_6\right) \tag{38}$$
$$\alpha_5 = \kappa_2 \kappa_5 \left(\kappa_4 + \kappa_6\right). \tag{39}$$

We use the expressions for α_1, α_3, α_4 and α_5 and solve for $\kappa_1, \kappa_3, \kappa_5, K_{\text{tot}}$ to obtain

$$K_{\text{tot}} = \frac{\alpha_1}{(\kappa_2 + 1)\, \alpha_3} \qquad \kappa_3 = \frac{\alpha_4 \kappa_2 \left(\kappa_4 + \kappa_6\right)}{\kappa_2^2 \kappa_4 \kappa_6 + \kappa_2^2 \kappa_6^2 + \kappa_2 \alpha_5 + \kappa_6 \alpha_5}$$
$$\kappa_1 = \frac{\alpha_3 \left(\kappa_2^2 \kappa_4 \kappa_6 + \kappa_2^2 \kappa_6^2 + \kappa_2 \alpha_5 + \kappa_6 \alpha_5\right)}{\alpha_4 \kappa_2 \kappa_6 \left(\kappa_4 + \kappa_6\right)} \qquad \kappa_5 = \frac{\alpha_5}{\kappa_2 \left(\kappa_4 + \kappa_6\right)}.$$

We plug these expressions into the equation for α_2 and obtain the equation

$$\alpha_2 = \frac{\alpha_1 \alpha_4 \alpha_5 \kappa_6}{\alpha_3 \left(\kappa_2^2 \kappa_4 \kappa_6 + \kappa_2^2 \kappa_6^2 + \kappa_2 \alpha_5 + \kappa_6 \alpha_5\right)} + \frac{(\kappa_4 + 1)\, \alpha_1 \kappa_2 \alpha_4 \alpha_5}{(\kappa_2 + 1)\, \alpha_3 \left(\kappa_2^2 \kappa_4 \kappa_6 + \kappa_2^2 \kappa_6^2 + \kappa_2 \alpha_5 + \kappa_6 \alpha_5\right)},$$

which is equivalent to the polynomial equation

$$\begin{aligned} 0 = &\left(-\kappa_2^3 \alpha_2 \alpha_3 - \kappa_2^2 \alpha_2 \alpha_3\right) \kappa_6^2 \\ &+ \left(-\kappa_2^3 \kappa_4 \alpha_2 \alpha_3 - \kappa_2^2 \kappa_4 \alpha_2 \alpha_3 + \kappa_2 \alpha_1 \alpha_4 \alpha_5 - \kappa_2 \alpha_2 \alpha_3 \alpha_5 + \alpha_1 \alpha_4 \alpha_5 - \alpha_2 \alpha_3 \alpha_5\right) \kappa_6 \\ &- \kappa_2^2 \alpha_2 \alpha_3 \alpha_5 + \kappa_2 \kappa_4 \alpha_1 \alpha_4 \alpha_5 + \kappa_2 \alpha_1 \alpha_4 \alpha_5 - \kappa_2 \alpha_2 \alpha_3 \alpha_5. \end{aligned}$$

The right-hand side of the equation can be seen as a polynomial of degree 2 in κ_6 with negative leader term. If the independent term is positive, then the polynomial has one positive root,

which determines the value of κ_6. Hence, we want to show that there exist values of κ_2, κ_4 such that

$$-\kappa_2\alpha_5\left(\kappa_2\alpha_2\alpha_3 - \kappa_4\alpha_1\alpha_4 - \alpha_1\alpha_4 + \alpha_2\alpha_3\right) > 0$$

or equivalently, that

$$\kappa_2\alpha_2\alpha_3 - \kappa_4\alpha_1\alpha_4 - \alpha_1\alpha_4 + \alpha_2\alpha_3 < 0.$$

For a fixed value of κ_2, this expression is a decreasing linear function on κ_4. Therefore, we can find a positive value of κ_4 such that it is negative.

We conclude that for any $\kappa_2 > 0$, we can find values of $\kappa_1, \kappa_3, \kappa_4, \kappa_5, \kappa_6 > 0$ and $K_{\text{tot}} > 0$ satisfying (35)-(39). □

As a consequence of Lemma 1, there exist values of the reaction rate constants and total amounts such that the network has $2n+1$ positive steady states if we can find $\alpha_{i,1}, \dots, \alpha_{i,5} > 0$ such that

$$0 = x - S_{\text{tot}} + \sum_{i=1}^{n} \frac{\alpha_{i,1}x^2 + \alpha_{i,2}x}{\alpha_{i,3}x^2 + \alpha_{i,4}x + \alpha_{i,5}} \tag{40}$$

has $2n+1$ positive solutions.

With this notation, we want to determine the number of positive roots of the polynomial obtained by clearing denominators in (40):

$$p(x) = (x - S_{\text{tot}})\prod_{i=1}^{n}(\alpha_{i,3}x^2 + \alpha_{i,4}x + \alpha_{i,5}) + \sum_{i=1}^{n}\Big((\alpha_{i,1}x^2 + \alpha_{i,2}x)\prod_{j\neq i}(\alpha_{j,3}x^2 + \alpha_{j,4}x + \alpha_{j,5})\Big). \tag{41}$$

The coefficient of degree $2n+1$ of $p(x)$ is $\prod_{i=1}^{n}\alpha_{i,3}$ and the independent term of $p(x)$ is $-S_{\text{tot}}\prod_{i=1}^{n}\alpha_{i,5}$. We set $\alpha_{i,4} = 0$ and $\alpha_{i,1} = 0$. Setting these two constants to zero, for $i = 1, \dots, n$, does not change the degree of the polynomial. By the continuity of the isolated roots of a polynomial as functions of the coefficients of the polynomial, if we can find $\alpha_{i,2}, \alpha_{i,3}, \alpha_{i,5} > 0$ such that with $\alpha_{i,4} = \alpha_{i,1} = 0$, the polynomial $p(x)$ has $2n+1$ distinct positive real roots, then for $\alpha_{i,4}, \alpha_{i,1}$ small enough, the polynomial $p(x)$ still has $2n+1$ distinct positive real roots.

We further let $\alpha_{i,3} = 1$ for all $i = 1, \dots, n$, and $S_{\text{tot}} = 1$. To ease the notation, we write $a_i = \alpha_{i,2}$ and $b_i = \alpha_{i,5}$, such that the polynomial of interest becomes

$$p(x) = (x-1)\prod_{i=1}^{n}(x^2 + b_i) + \sum_{i=1}^{n}\Big(a_i x \prod_{j\neq i}(x^2 + b_j)\Big). \tag{42}$$

Lemma 2. *There exist $a_i, b_i > 0$, for $i = 1, \dots, n$, such that $p(x)$ has $2n+1$ positive roots.*

Proof. The statement is a consequence of Lemmas 2, 3 and 4 in the Supplementary Information of [7]. □

We are ready to prove the main result on the number of positive steady states.

Theorem 13. *For any $n \geq 1$, there exists a choice of reaction rate constants and total amounts such that the reduced network with n allosteric kinases competing for the same substrate has $2n+1$ distinct positive steady states.*

Proof. Let $b_i, a_i > 0$, for $i = 1, \ldots, n$, as in Lemma 2, such that $p(x)$ in (42) has $2n + 1$ distinct positive real roots. We set $\alpha_{i,2} = a_i, \alpha_{i,5} = b_i, \alpha_{i,3} = 1$, for $i = 1, \ldots, n$, $S_{\text{tot}} = 1$ and let $\alpha_{i,1}, \alpha_{i,4} > 0$ be small enough such that the polynomial $p(x)$ in (41) has $2n + 1$ distinct positive real roots. We set $k_7 = 1$. By construction, any choice $\kappa_{i,1}, \ldots, \kappa_{i,6} > 0$ and $K^i_{\text{tot}} > 0$ such that (30)-(34) are fulfilled provides a set of parameters with $2n + 1$ distinct positive steady states. Such a choice exists by Lemma 1. □

Remark 14 (Existence of $2m + 1$ steady states)**.** Consider the polynomial $p(x)$ in (42) and let $a_k = 0$ for a certain k. We obtain the polynomial

$$\begin{aligned}\tilde{p}(x) &= (x-1)\prod_{i=1}^{n}(x^2+b_i) + \sum_{i=1, i\neq k}^{n}\Big(a_i x \prod_{j\neq i}(x^2+b_j)\Big)\\ &= (x^2+b_k)\cdot\Big((x-1)\prod_{i=1, i\neq k}^{n}(x^2+b_i) + \sum_{i=1, i\neq k}^{n}\Big(a_i x\prod_{j\neq i,k}(x^2+b_j)\Big)\Big).\end{aligned}$$

Assuming $b_k > 0$, the polynomial $\tilde{p}(x)$ factorises into two factors: one factor of degree two with non-real roots and the other factor is of the form of (42) with degree $2(n-1)+1 = 2n-1$. By Lemma 2, the latter factor admits $2n-1$ positive roots for some choice of a_i, b_i. Therefore, we conclude that $\tilde{p}(x)$ admits precisely $2n - 1$ positive roots for some choice of parameters as well. By the continuity of the roots of a polynomial, this remains true for $a_k > 0$ small enough. Proceeding as in the proof of Theorem 13, this implies that there exist reaction rate constants and total amounts such that the system has precisely $2n - 1$ positive steady states.

We can repeat the argument by letting m of the parameters among $a_1, \ldots, a_n$ be equal to zero, and conclude that we can find reaction rate constants and total amounts such that the system has precisely $2m + 1$ positive steady states for all $m = 0, \ldots, n$.

2.5 n unstable steady states

In this subsection we show that, considering the $2n + 1$ steady states ordered increasingly by their value of x_5, then the steady states number $2, 4, \ldots, 2n$ are unstable relative to the invariant subspaces described by the conservation laws (22) and (23).

Consider the Jacobian $J_f(x)$ of the function f in the ODE system $\dot{x} = f(x)$. Since the system with n allosteric kinases competing for the same substrate has $4n + 2$ variables and $n+1$ conservation laws, the Jacobian of f in the ODE system $\dot{x} = f(x)$ always has $n+1$ zero eigenvalues. The eigenvectors of the remaining $3n + 1$ eigenvalues (which could include zero) belong to the so-called stoichioimetric subspace, which has equations

$$x_{i,1} + x_{i,2} + x_{i,3} + x_{i,4} = 0, \quad i = 1, \ldots, n, \qquad x_5 + x_6 + \sum_{i=1}^{n}(x_{i,3} + x_{i,4}) = 0,$$

and dictate the dynamics around the steady state within this space. If the steady state is locally asymptotically stable relative to the invariant subspace described by the conservation laws, then the product of these $3n + 1$ eigenvalues has sign $(-1)^{3n+1}$. Therefore, if the sign of the product of these eigenvalues is $(-1)^{3n}$, then the steady state is necessarily unstable relative to the invariant subspace. We argue in the proof of the next theorem that this is the case for the steady states in even position $2, 4, \ldots, 2n$.

Theorem 15. *The $2, 4, \ldots, 2n$-th steady states are unstable relative to the invariant subspace described by the conservation laws.*

Proof. We order the variables of the system as

$$x = (x_{1,1}, x_{1,2}, x_{1,3}, x_{1,4}, \dots, x_{n,1}, x_{n,2}, x_{n,3}, x_{n,4}, x_5, x_6).$$

It follows from [8, Prop. 5.3] that the product of the $3n+1$ eigenvalues of the Jacobian with eigenvectors in the invariant subspace agrees with the determinant of the Jacobian of the function $g\colon \mathbb{R}^{4n+2} \to \mathbb{R}^{4n+2}$, where

$$\begin{aligned}
g_{4(i-1)+1}(x) &= x_{i,1} + x_{i,2} + x_{i,3} + x_{i,4} - K^i_{\text{tot}} \\
g_{4(i-1)+2}(x) &= -\kappa_{i,3} x_{i,2} x_5 + \kappa_{i,4} x_{i,4} + \kappa_{i,5} x_{i,1} \\
g_{4(i-1)+3}(x) &= \kappa_{i,1} x_{i,1} x_5 - \kappa_{i,2} x_{i,3} + \kappa_{i,6} x_{i,4} \\
g_{4(i-1)+4}(x) &= \kappa_{i,3} x_{i,2} x_5 - \kappa_{i,4} x_{i,4} - \kappa_{i,6} x_{i,4}
\end{aligned}$$

for $i = 1, \dots, n$ and

$$g_{4n+1}(x) = x_5 + x_6 + \sum_{j=1}^{n} (x_{j,3} + x_{j,4}) - S_{\text{tot}},$$

$$g_{4n+2}(x) = \sum_{j=1}^{n} (\kappa_{j,2} x_{j,3} + \kappa_{j,4} x_{j,4}) - \kappa_7 x_6.$$

Therefore, the steady state is unstable if the sign of the determinant of the Jacobian of g is $(-1)^{3n}$.

We now apply the method described in [3] (see the Electronic Supplementary Material of the paper), to determine the sign of the determinant of the Jacobian of g from iterative eliminations. One can check that the expressions in (24)-(27) are obtained from iteratively eliminating $x_{i,1}, \dots, x_{i,4}$ from the equations $g_{4(i-1)+1}(x) = \dots = g_{4(i-1)+4}(x) = 0$ respectively, which correspond to the conservation law with total amount K^i_{tot} together with $\dot{x}_{i,2} = \dot{x}_{i,3} = \dot{x}_{i,4} = 0$. For each i, we first eliminate $x_{i,1}$ from $g_{4(i-1)+1}(x) = 0$. We have that $g_{4(i-1)+1}(x)$ increases in $x_{i,1}$. We substitute next the value of $x_{i,1}$ into $g_{4(i-1)+2}(x)$. The resulting expression decreases in $x_{i,2}$. We isolate $x_{i,2}$ then from the equation $g_{4(i-1)+2}(x) = 0$. We proceed in the same way to find $x_{i,3}$ and $x_{i,4}$ from $g_{4(i-1)+3}(x) = 0$ and $g_{4(i-1)+4}(x) = 0$. The functions $g_{4(i-1)+3}(x)$ and $g_{4(i-1)+4}(x)$ (after the appropriate substitutions are made) are decreasing in $x_{i,3}$ and $x_{i,4}$ respectively. Finally, x_6 is eliminated from $g_{4n+2}(x) = 0$. Note that $g_{4n+2}(x)$ decreases in x_6.

Let $p(x_5)$ be the polynomial obtained after clearing denominators in (29). Then, by [3, Proposition 7], the sign of the determinant of the Jacobian of g at a steady state agrees with the sign of the derivative of $p(x_5)$, $p'(x_5)$, times $(-1)^{3n+1}$. Therefore, if $p'(x_5)$ is negative, then the corresponding steady state is unstable. Since $p(0)$ is negative, the first real root of $p(x_5)$ has positive derivative, and then the signs alternate. Therefore, the steady states corresponding to the $2, 4, \dots, 2n$-th roots are unstable relative to the invariant subspace described by the conservation laws. □

3 Allosteric kinases with several states

In this section we consider the case in which the allosteric kinase is specific to one substrate, but the kinase might have more than 2 states and transitions between all states occur. Let K_i, $i = 1, \dots, n$ denote the n states of the kinase. The general model is in this case:

$$K_i + S \rightleftharpoons K_iS \rightarrow K_i + S_p \qquad S_p \rightarrow S \qquad K_i \rightleftharpoons K_j \qquad K_iS \rightleftharpoons K_jS$$

for all $i, j = 1, \dots, n$, $i \neq j$.

In order to analyse this system, we consider a reduced model. Then we use Theorem 1 and Theorem 2 to translate results on the number of positive steady states of the reduced model to the model of interest. Explicitly, we consider the cases where $n = 3$ and $n = 4$.

We proceed through three simplification steps to obtain the reduced network.

(i) We make the reversible reactions $K_i + S \rightleftharpoons K_iS$ irreversible, that is, we consider only the reaction $K_i + S \longrightarrow K_iS$.

(ii) We set the reaction rate constants of $K_iS \to K_jS$ and $K_j \to K_i$ to zero except for $j = i - 1$. That is, the only conformational changes we consider in the model are

$$K_1 \to K_2 \to \dots \to K_n \qquad\qquad K_nS \to K_{n-1}S \to \dots \to K_1S$$

(iii) We remove the intermediates K_iS for $i = n, \dots, 2$ iteratively, as explained in subsection 1.3. That is, we remove K_nS, then $K_{n-1}S$ etc. We denote the only intermediate that is left by $Y := K_1S$. The reduced model is

$$\begin{array}{llll} K_i + S \to Y & \text{for } i = 1, \dots, n & Y \to K_1 + S_p & \\ K_i + S \to K_j + S_p & \text{for } i \geq j \geq 2 & K_1 \to K_2 \to \dots \to K_n & S_p \to S. \end{array}$$

To summarise, the reduced model we consider is:

$$\begin{array}{llll} K_i + S \xrightarrow{\kappa_i} Y & \text{for } i = 1, \dots, n & Y \xrightarrow{\eta_1} K_1 + S_p & \\ K_i + S \xrightarrow{\mu_{i,j}} K_j + S_p & \text{for } i \geq j \geq 2 & K_1 \xrightarrow{\eta_2} K_2 \xrightarrow{\eta_3} \dots \xrightarrow{\eta_n} K_n & S_p \xrightarrow{\kappa_{n+1}} S. \end{array}$$

When $n = 3$, the reduced model cannot have more than 3 positive steady states: by proceeding as in subsection 1.4, we find that the positive steady states are in one-to-one correspondence with the positive roots of a degree 4 polynomial with positive independent term and negative leading term. By the Descartes' rule of sign, the polynomial can at most have 3 positive real roots.

For $n = 4$, we find the following instance of parameters that give 5 positive steady states:

$$\begin{array}{lllll} \kappa_1 = 0.0369, & \kappa_2 = 0.000172, & \kappa_3 = 1.4 \cdot 10^{-9}, & \kappa_4 = 0.00011, & \kappa_5 = 1069.496, \\ \mu_{2,2} = 0.0003, & \mu_{3,2} = 0.000008426, & \mu_{4,2} = 0.00016, & K_{\text{tot}} = 102, & \\ \mu_{3,3} = 0.000085, & \mu_{4,3} = 1999.97, & \mu_{4,4} = 25165410, & S_{\text{tot}} = 120, & \\ \eta_1 = 0.0107, & \eta_2 = 0.1, & \eta_3 = 0.001, & \eta_4 = 0.1. & \end{array}$$

By Theorem 1 and Theorem 2, we conclude that the full model with an allosteric kinase with n states also admits 5 positive steady states for some choice of reaction rate constants and total amounts.

The general scenario is too complex mathematically and cannot be analysed as we did for the model in section 2. We conjecture though that the number of positive steady states grows with n as well, such that the system admits at least $m + 1$ positive steady states where $m = n$ if n is even and $m = n - 1$ if n is odd.

References

[1] C. Conradi, E. Feliu, M. Mincheva, and C. Wiuf. Identifying parameter regions for multistationarity. *ArXiv.org*, (1608.03993), 2016.

[2] C. Conradi and M. Mincheva. Catalytic constants enable the emergence of bistability in dual phosphorylation. *J. R. S. Interface*, 11(95):20140158–20140158, March 2014.

[3] E. Feliu and C. Wiuf. Enzyme-sharing as a cause of multi-stationarity in signalling systems. *J. R. S. Interface*, 9(71):1224–32, 2012.

[4] E. Feliu and C. Wiuf. Simplifying biochemical models with intermediate species. *J. R. S. Interface*, 10:20130484, 2013.

[5] E. Feliu and C. Wiuf. Finding the positive feedback loops underlying multi-stationarity. *BMC Systems Biology*, 9, 2015.

[6] B. Joshi and A. Shiu. Atoms of multistationarity in chemical reaction networks. *J. Math. Chem.*, 51(1):153–178, 2013.

[7] V. B. Kothamachu, E. Feliu, L. Cardelli, and O. S. Soyer. Unlimited multistability and boolean logic in microbial signalling. *J. R. S. Interface*, 12(108), 2015.

[8] C. Wiuf and E. Feliu. Power-law kinetics and determinant criteria for the preclusion of multistationarity in networks of interacting species. *SIAM J. Appl. Dyn. Syst.*, 12:1685–1721, 2013.
