# Supplementary figures and images for: Core signalling motif displaying multistability through multi-state enzymes
Source: J R Soc Interface. 2016 Oct;13(123):20160524. doi: 10.1098/rsif.2016.0524 (PMC5095215; doi:10.1098/rsif.2016.0524)

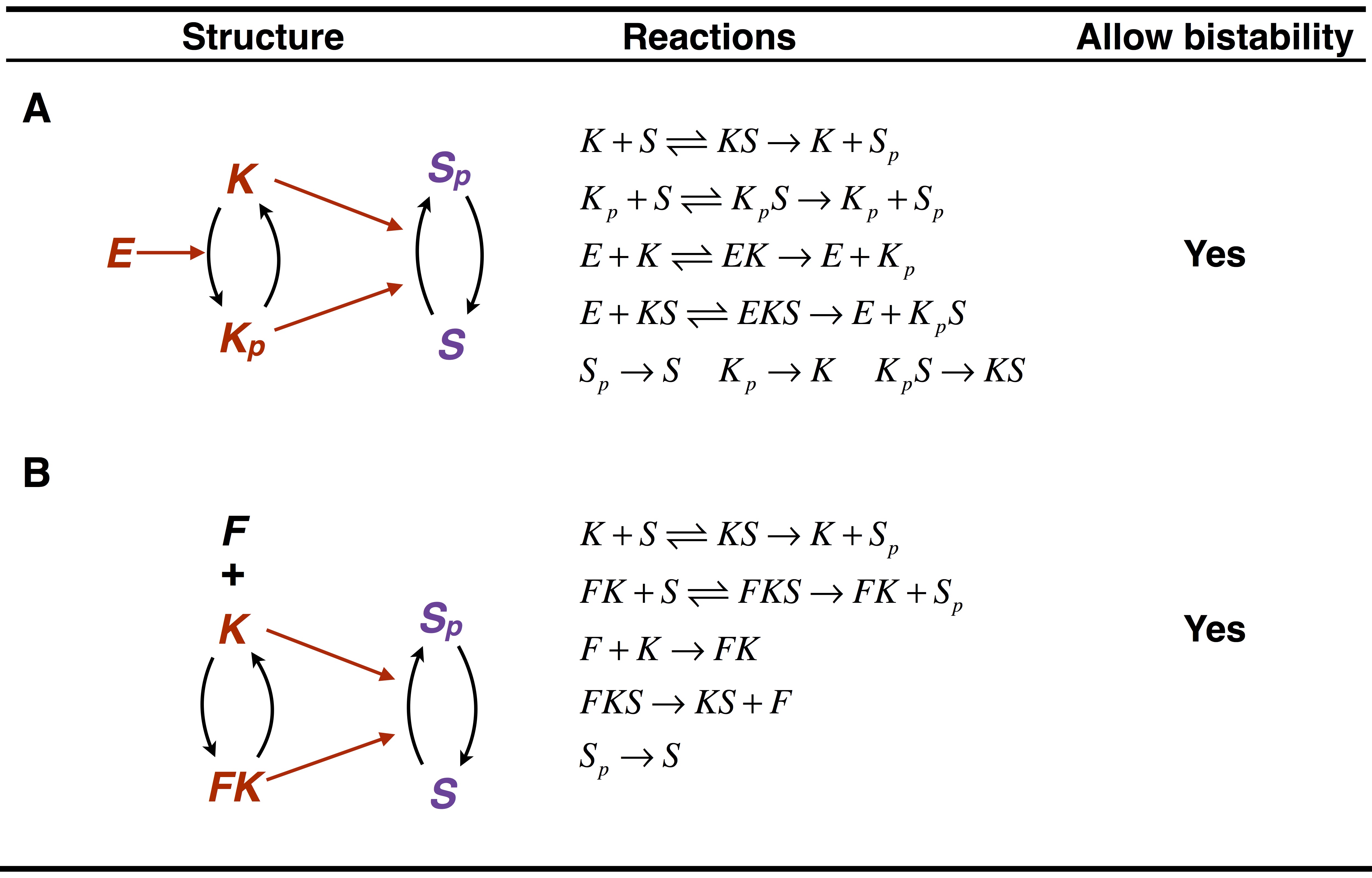

Supplement: Supplementary Figure [file rsif20160524supp2.jpg]

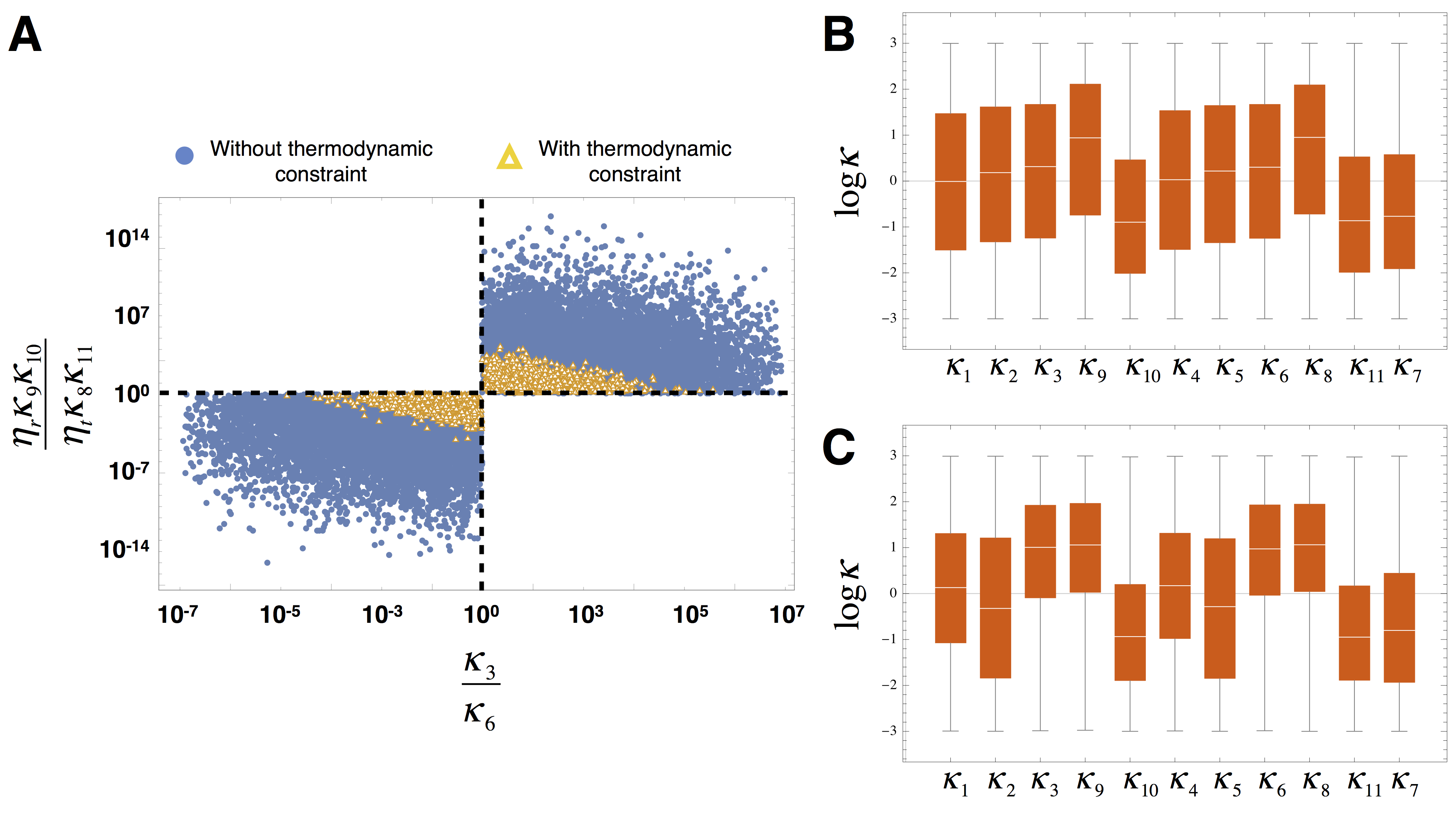

Supplement: Supplementary Figure [file rsif20160524supp3.tiff]
